# A Novel Improved Dung Beetle Optimization Algorithm for Collaborative 3D Path Planning of UAVs

**DOI:** 10.3390/biomimetics10070420

**Published:** 2025-06-29

**Authors:** Xiaojun Zheng, Rundong Liu, Siyang Li

**Affiliations:** School of Mechanical Engineering, Dalian Jiaotong University, Dalian 116028, China; 15589542303@163.com (R.L.); 15041194309@163.com (S.L.)

**Keywords:** dung beetle optimizer, improvement strategies, CEC2017, path planning, cooperative UAVs

## Abstract

In this study, we propose a novel improved Dung Beetle Optimizer called Environment-aware Chaotic Force-field Dung Beetle Optimizer (ECFDBO). To address DBO’s existing tendency toward premature convergence and insufficient precision in high-dimensional, complex search spaces, ECFDBO integrates three key improvements: a chaotic perturbation-based nonlinear contraction strategy, an intelligent boundary-handling mechanism, and a dynamic attraction–repulsion force-field mutation. These improvements reinforce both the algorithm’s global exploration capability and its local exploitation accuracy. We conducted 30 independent runs of ECFDBO on the CEC2017 benchmark suite. Compared with seven classical and novel metaheuristic algorithms, ECFDBO achieved statistically significant improvements in multiple performance metrics. Moreover, by varying problem dimensionality, we demonstrated its robust global optimization capability for increasingly challenging tasks. We further conducted the Wilcoxon and Friedman tests to assess the significance of performance differences of the algorithms and to establish an overall ranking. Finally, ECFDBO was applied to a 3D path planning simulation in UAVs for safe path planning in complex environments. Against both the Dung Beetle Optimizer and a multi-strategy DBO (GODBO) algorithm, ECFDBO met the global optimality requirements for cooperative UAV planning and showed strong potential for high-dimensional global optimization applications.

## 1. Introduction

Unmanned aerial vehicles (UAVs) have experienced rapid development and have been widely deployed in various domains since their inception. UAVs have been applied in such specialized areas as soil information collection and irrigation decision support in agriculture [1], deterring birds, connecting nodes in communication networks, monitoring the marine environment [2], and urban planning [3]. Three-dimensional (3D) path planning is a critical component of autonomous UAV missions, as it directly determines whether a UAV can navigate complex environments safely and efficiently to reach its destination [4]. An optimized flight path must not only avoid terrain obstacles but also minimize travel distance while adhering to the dynamic constraints of the aircraft. These requirements make path planning particularly challenging in 3D spaces with complex terrain and numerous static obstacles [5]. It is also essential to consider flight altitude, camera angle, overlap rate, and obstacle avoidance to enable comprehensive 3D observation and accurate modeling, especially in areas with varying topography or dense urban structures [6,7,8,9].

Among the classical global path planning algorithms, the grid-based search methods (e.g., A*) are capable of finding the optimal paths in static environments, but they incur large memory overhead and are sensitive to the environment scale, making them unsuitable for planning in vast 3D space [10,11,12,13,14,15,16]. The artificial potential field methods are highly respected for their simplicity and computational speed, and have been used for UAV obstacle avoidance; however, pure potential field methods do not guarantee global optimality and are prone to falling into local minima in complex terrains, leading the path to ‘deadlocks’ [17,18]. Another class of sampling-based expansion algorithms, namely Rapidly exploring Random Trees (RRT) and its improved variant RRT*, can effectively expand paths in continuous space, but RRT makes it difficult to strike a balance between path optimality and obstacle safety, thus may still produce excessively long or non-smooth paths [19]. To address these problems, researchers have proposed various improvements to RRT; for example, Guo et al. introduced a fuzzy control strategy to optimize the sampling process and developed the FC-RRT* algorithm to improve search efficiency and safety in complex 3D environments [20]. In conclusion, traditional algorithms often face such problems as high computational costs and being prone to falling into suboptimal solutions in challenging terrains and scenarios with many obstacles.

In recent years, intelligent optimization algorithms have been increasingly applied in UAV path planning owing to their strong global search capabilities [21]. Genetic algorithms (GA), which are naturally adept at multi-objective optimization, have been employed in planning UAV paths under multiple tasks or constraints. For example, Pehlivanoglu et al. devised an improved GA for UAV coverage missions [22]. Particle Swarm Optimization (PSO) algorithms [23,24] have gained widespread attention because of their fast convergence and small number of parameters. Researchers have proposed numerous variants tailored to UAV path-planning needs, including phase-angle-encoded PSO [25,26,27], quantum-behaved PSO [28], and discrete PSO [29,30]. Differential evolution (DE) algorithms excel in global search capability, and some scholars have proposed hybrid DE algorithms in combination with strategies such as grey wolf optimization to plan 3D paths in complex mountainous terrain [31]. Additionally, swarm intelligence methods like Ant Colony Optimization (ACO) [32] and Artificial Bee Colony (ABC) [33] have been applied to UAV obstacle-avoidance path planning. However, single-strategy meta-heuristics often suffer from getting trapped in local optima or lacking sufficient convergence precision. Original PSO, for instance, is prone to converge prematurely, while ACO’s communication-based modeling incurs substantial computational overhead, limiting its real-time application [34]. In addition, researchers have explored various types of plants, animals, and insects in nature and invented various meta-heuristic algorithms. Inspired by the foraging behavior of coatis, Dehghani M et al. proposed the Coati Optimization Algorithm (COA) [35]. Arora S et al. modeled the food search and mating behavior of butterflies and proposed and tested the Butterfly Optimization Algorithm (BOA) [36] based on the foraging strategy of butterflies. Zhong et al. proposed the Beluga Whale Optimization (BWO) algorithm, inspired by the collective behavior of beluga whales [37]. Duan and Qiao introduced the Pigeon-Inspired Optimization (PIO) algorithm, based on the navigational behavior of pigeon flocks. PIO has been successfully applied to flight-robot path planning [38]. Recently, the Nutcracker Optimization Algorithm (NOA) was proposed, simulating the spatial memory and random foraging behavior of nutcracker birds in their seed search, caching, and retrieval process [39]. Liu and Cai et al. proposed a hybrid multi-strategy artificial rabbit optimization (HARO) [40]; by simulating spherical and cylindrical obstacle models, they achieved efficient and stable UAV path planning in complex environments. Therefore, these advancements highlight an emerging research focus: refining algorithms to meet the specific demands of UAV path planning by striking a more effective balance between global exploration capability and local optimization precision.

In response to the above optimization requirements, Dung Beetle Optimizer (DBO) offers a novel perspective for UAV path planning. First proposed by Xue and Shen in 2022 [41], DBO draws inspiration from the behavioral patterns of dung beetles, incorporating five typical behavioral mechanisms—rolling, dancing, foraging, stealing, and reproducing. When applied to three examples of engineering optimization, DBO also achieved superior results compared to benchmark algorithms; the potential of it for practical applications is further confirmed. Owing to these advantages, DBO has attracted increasing attention in the field of path planning and is considered a promising approach for solving complex optimization tasks such as 3D UAV path planning.

Despite the strong performance of the original DBO, subsequent research has revealed certain limitations when the algorithm is applied to high-dimensional and complex search spaces. Responding to these shortcomings, scholars have proposed several enhanced variants of the Dung Beetle Optimizer. For single-UAV path planning, Shen et al. introduced the Multi-strategy Dung Beetle Optimizer (MDBO) [42]. In 2024, Tang et al. proposed RCDBO, an enhanced DBO variant for robotic path planning [43]. Another notable variant is GODBO, introduced by Wang et al. [44], which enhances exploration through opposition-based learning and multiple strategies centered on the current best solution. Results indicated clear improvements in both convergence precision and speed. Similarly, SSTDBO, proposed by Hu et al. [45], was designed to overcome the original DBO’s deficiencies in population diversity, global detection ability, and convergence precision.

For multi-UAV cooperative planning, Zhang et al. proposed the Multi-strategy Improved Dung Beetle Optimizer (MIDBO) for UAV task allocation [46]. In another study, Shen Q. et al. extended DBO to the multi-objective optimization domain by introducing the Directed Evolution Non-dominated Sorting Dung Beetle Optimizer (DENSDBO-ASR) [47]. This algorithm was applied to cooperative multi-UAV path planning and achieved excellent results in both convergence precision and solution set diversity. Collectively, these enhancements demonstrate that integrating chaos theory, evolutionary operators, and distribution-based mechanisms can substantially boost the performance of DBO, making it more suitable for high-dimensional and complex path-planning tasks.

Many researchers have proposed various DBO improvement algorithms, such as MDBO, SSTDBO, and GODBO. However, there are still some problems that need to be solved in high-dimensional, complex search spaces and in actual UAV 3D path planning tasks. For example, SSTDBO can maintain population diversity in the early stages of the search, but it easily falls into local optima due to the fast convergence of strategies, especially in scenarios with more than 50 dimensions. The premature convergence of the algorithm is especially obvious. Second, MDBO is prone to premature population convergence in high-dimensional, complex environments, making it difficult to escape local optima. Furthermore, GODBO enhances exploration capability by applying multi-strategy learning to the current optimal solution. However, it struggles to escape local optimization in multi-constraint UAVs. GODBO enhances exploration capability by applying multi-policy learning to the current optimal solution. However, it exhibits high computational overhead in multi-constraint UAV tasks, affecting the algorithm’s real-time performance and practicality.

In summary, the Dung Beetle Optimizer offers both advantages and limitations in UAV path planning. On the one hand, DBO integrates diverse biological behavior mechanisms, which makes DBO have strong global exploration and local exploitation capabilities. Particularly, strategy-enhanced variants of DBO have shown improved adaptability to complex terrain, enabling safer and more efficient path planning in large-scale 3D environments [48]. On the other hand, the original DBO is still prone to premature convergence and becoming trapped in local optima. In environments with complex obstacle distributions, this can lead to insufficient search diversity and difficulty escaping suboptimal regions [49]. Furthermore, as UAV path planning is inherently a high-dimensional optimization problem, the computational cost of DBO increases with problem scale, making it essential to balance convergence speed and algorithm efficiency in practical applications. For these challenges, we propose a novel improved DBO variant for 3D UAV path planning in environments with complex terrain and static obstacles. The algorithm integrates three core strategies: nonlinear contraction of chaotic perturbation, which significantly inhibits premature convergence; an intelligent boundary-handling mechanism that realizes an “energy wall” rebound at the obstacle boundary in a gradient-guided manner to improve the algorithm’s ability to adapt to a dynamic environment; and an attraction-repulsion force-field variability strategy that adjusts adaptively with iterations. This strategy effectively balances global search and local development, resulting in better convergence accuracy and computational efficiency for high-dimensional complex problems and multi-constraint UAV path planning. This study makes the following specific contributions:

1. An Environment-aware Chaotic Force–field Dung Beetle Optimizer (ECFDBO) is proposed by us, which augments the original Dung Beetle Optimizer (DBO) with three novel improvement strategies: chaotic perturbation mixed nonlinear contraction, environment-aware boundary handling, and dynamic attraction–repulsion field mutation. It ensures stable performance in high-dimensional search spaces.
2. ECFDBO was subjected to multi-perspective, multi-run experiments by us on the CEC2017 (Dim = 30, 50, and 100) suite to assess its robustness and effectiveness. Wilcoxon and Friedman’s tests demonstrate that ECFDBO’s performance differences among seven state-of-the-art metaheuristics are statistically significant.
3. We formulate the multi-UAV coordination task as a multi-constrained optimization problem with several key constraints based on the application of UAV tasks in remote sensing. Additionally, we apply the cooperative path-planning model to four distinct environments to validate its simulated effectiveness.

The rest of the paper is structured as follows. Section 2 reviews the preliminary knowledge of the original DBO algorithm. Section 3 details the three novel improvement mechanisms introduced in ECFDBO. Section 4 analyzes the computational complexity of the proposed algorithm. Section 5 presents benchmark results on the CEC2017 suite and accompanying statistical tests. Section 6 describes the UAV simulation experiments and discusses the results. Section 7 concludes the paper and outlines directions for future work.

## 2. Preliminary Knowledge

**Figure 1:**
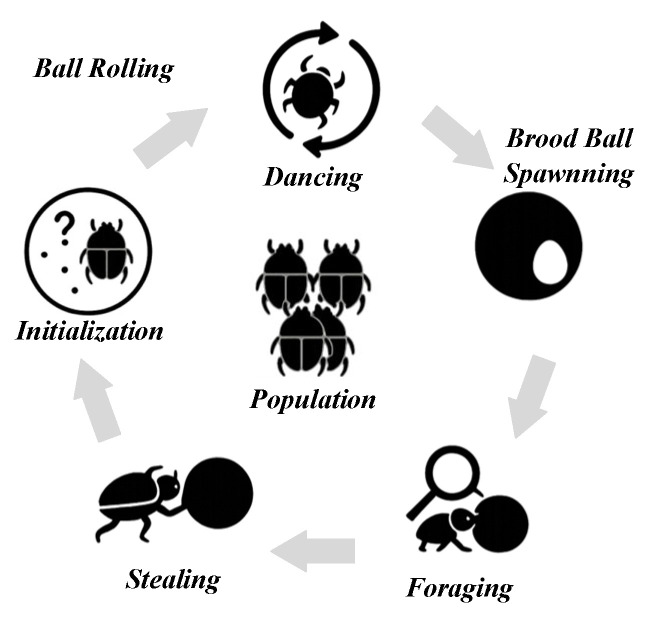
Dung Beetle Optimizer.

The DBO algorithm is derived from the survival behavior of dung beetles in their natural environment. The algorithm plays an important role in achieving global dominance by simulating the rolling of a dung ball by a beetle and the subsequent redirection of movement by implementing a dancing behavior. The beetles adopt this behavior when encountering obstacles, thus preventing falling into local traps. Female dung beetles establish a localized exploration area by hiding dung balls and laying eggs. Following this initial foraging behavior, the young dung beetles mimic the adults and thus perform a fine search in a small area. Finally, competitive behavior occurs between individuals, accelerating the approach to the optimal solution. This phenomenon is illustrated in Figure 1.

### 2.1. Population Initialization

The Dung Beetle Optimizer initializes its population in a randomized manner, where each dung beetle’s position corresponds to a potential solution to the optimization problem. For the d dimensional optimization problem, the position of the i-th dung beetle is denoted as $x_{i}=\left[x_{i,1},x_{i,2},\ldots ,x_{i,d}\right]$, and the population of dung beetles of N size can be denoted as $X={\left[x_{1},x_{2},\ldots ,x_{N}\right]}^{T}$, as shown in Equation (1).

(1) $$X=\left[\begin{array}{ccc} x_{1,1} & \ldots & x_{1,d}\\ \vdots & \ddots & \vdots \\ x_{N,1} & \ldots & x_{N,d} \end{array}\right]$$

### 2.2. Rolling Stage

#### 2.2.1. Obstacle-Free Mode

In the absence of obstacles, a rolling dung beetle simulates its natural counterpart by carrying a dung ball multiple times its size on its back and moving in a straight line in flat or slightly undulating terrain with the help of sun or moonlight. This phase focuses on “covering a wider search space with large steps.” The ball’s position is updated according to Equation (2).

(2) $$x_{i}(t+1)=x_{i}(t)+\phi \cdot k\cdot x_{i}(t-1)+b\Delta x$$

where $\phi \cdot k\cdot x_{i}(t-1)$ simulates the inertia of the dung beetle continuing in its original direction, ϕ=±1 is randomly selected to reflect small deflections in the rolling direction due to natural disturbances such as wind and terrain, $\Delta x=|x_{i}(t)-X_{w}|$, $X_{w}$ are the current worst individual locations, which encourages the dung beetle to move away from the inferior region by increasing the distance to the worst solution, and $x_{i}(t)$ maintains the continuity of the algorithm in the current optimal neighborhood.

#### 2.2.2. Obstacle Mode

When a dung beetle encounters an obstacle during rolling and cannot continue in its original direction, it performs a “dancing” behavior—rotating on its dung ball and pausing momentarily to recalibrate its path. The position update rule corresponding to this dancing behavior is defined in Equation (3).

(3) $$x_{i}(t+1)=x_{i}(t)+\tan(\theta )\left|x_{i}(t)-x_{i}(t-1)\right|$$

It has been demonstrated that the magnitude of the movement in the new direction is proportional to the difference between the two positions of the dung beetle before and after. Furthermore, the value of tan(θ) takes on a characteristic that produces diversity from zero deflection θ=0,π/2,π to large steering. This helps the algorithm to jump out of the trap and avoid falling into local optimization.

### 2.3. Reproduction Behavior

Female dung beetles will set aside a safe spawning area for their young within a certain distance from the main path of the colony—close to the food source but avoiding detection by predators. The algorithm dynamically defines the upper and lower boundaries of the spawning area centered on the current local optimal position $X^{*}$, as shown in Equation (4).

(4) $$\begin{array}{l} Lb^{*}=\max\left(X^{*}\times (1-R),Lb\right),\\ Ub^{*}=\min\left(X^{*}\times (1+R),Ub\right) \end{array}$$

where $R=1-t/T_{\max}$; Lb, Ub denote the lower and upper bounds of the optimization problem; as the iteration advances (R decreases gradually), the spawning area shrinks from a wide area to a narrow area, which is conducive to fine mining near the optimal solution. A single spawn location is generated per iteration, as shown in Equation (5).

(5) $$B_{i}(t+1)=X^{*}+b_{1}\times \left(B_{i}(t)-Lb^{*}\right)+b_{2}\times \left(B_{i}(t)-Ub^{*}\right)$$

where $B_{i}(t)$ is the position information of the i-th spawn ball at the t-th iteration; $b_{1}$ and $b_{2}$ are two independent random vectors of size 1×d. Egg balls are strictly confined to spawning areas.

### 2.4. Foraging Behavior

Some of the dung beetles leave the spawning area to forage on the ground, representing the algorithm’s diverse and smaller step-size fine search around the high-quality area. It also dynamically defines the foraging area centered on the global optimization $X^{b}$, as shown in Equation (6).

(6) $$\begin{array}{c} Lb^{b}=\max\left(X^{b}\times (1-R),Lb\right),\\ Ub^{b}=\min\left(X^{b}\times (1+R),Ub\right) \end{array}$$

where $Lb^{b}$, $Ub^{b}$ are the lower and upper bounds of the optimal foraging area, respectively. The position of the small dung beetle is updated by measuring normal perturbation and linear interpolation, as shown in Equation (7).

(7) $$x_{i}(t+1)=x_{i}(t)+C_{1}\times \left(x_{i}(t)-Lb^{b}\right)+C_{2}\times \left(x_{i}(t)-Ub^{b}\right)$$

where $C_{1}$ is a random number following a normal distribution; $C_{2}$ is a random vector in the range $\left(0,1\right)$.

### 2.5. Stealing Behavior

In high-density areas, some dung beetles attempt to “steal” dung balls from others to quickly acquire resources. In the algorithm, this behavior corresponds to a jump-based position update that drives individuals toward convergence on the global best solution. The position update rule for this stealing behavior is defined in Equation (8).

(8) $$x_{i}(t+1)=X^{b}+S\times g\times \left\{\left|x_{i}(t)-X^{*}\right|+\left|x_{i}(t)-X^{b}\right|\right\}$$

where g is a random vector of size 1×d obeying a normal distribution and S is a constant controlling the jump amplitude. Pseudo code such as the Algorithm 1.

The Pseudo-Code of DBO:


**Algorithm 1: DBO (Dung Beetle Optimizer)**
Input: population size: N; problem dimension: d; search boundary: [lb, ub]; maximum number of iterations: T_max_; fitness function: f(x) Output: Optimal position: X_best_1:      Initialize X_i_ ∼ Uniform(lb, ub) for i = 1…N ←Calculated using Equation (1) 2:      Evaluate f_i_ = f(X_i_), set X_best_ = argmin f_i_, X_worst_ = argmax f_i_ 3:      for t = 1 to T_max_ do 4:              p = ⌊0.2·N⌋ 5:              // Rolling stage 6:              for i = 1 to p do 7:                    if rand() < 0.9 then 8:                            X_i_ ←Calculated using Equation (2) 9:                    else 10:                          X_i_ ←Calculated using Equation (3) 11:                  end if 12:            end for 13:            // Reproduction stage 14:            R = 1 − t/T_max_ 15:            [Lb*, Ub*] ←Calculated using Equation (4) 16:            for i = p + 1 to p + m do 17:                  X_i_ ←Calculated using Equation (5) 18:            end for 19:            // Foraging stage 20:            for i = p + m+1 to p + m+q do 21:                  X_i_ ←Calculated using Equation (6) 22:            end for 23:            // Stealing stage 24:            for i = p + m+q + 1 to N do 25:                  X_i_ ←Calculated using Equation (7) 26:            end for 27:            // Boundary check 28:            for i = 1 to N do 29:                  X_i_ = clip(X_i_, lb, ub) 30:            end for 31:            Evaluate all f_i_, update X_best_, X_worst_ 32:    end for 33:    return X_best_

## 3. Proposed Algorithm

According to the No Free Lunch theorem [50], different algorithms perform variably on different problems, so there is a continual need for novel optimization strategies in academic research.

### 3.1. Chaotic Perturbation Mixed Nonlinear Contraction Mechanisms

In the ecological behavior of dung beetles, real individuals exhibit remarkable dynamic spatial adaptability. When a population cooperates around a core resource (the dung ball), its range of activity follows a spiral contraction pattern, gradually shrinking over time. This spatial focusing strategy creates a negatively correlated feedback mechanism with an individual’s energy reserves, causing their radius of motion to decrease at a nonlinear rate, showing a preferential guarding of vested resources and conservative local search behavior.

Inspired by this, a chaotic perturbation–based nonlinear contraction mechanism is designed to enhance the foraging behavior in the algorithm. The specific update rule is provided in Equation (9).

(9) $$X_{i}^{t+1}=X_{\mathrm{best}\;}^{t}+\Phi \left(r_{1}\right)\left(X_{\mathrm{best}\;}^{t}-X_{i}^{t}\right)+\Psi \left(r_{2}\right)\left(X_{\mathrm{cbest}\;}^{t}-X_{i-1}^{t}\right)$$

(10) $$\Psi \left(r_{2}\right)=tanh\left(\gamma r_{2}\right)\cdot e^{-\lambda r_{2}^{2}}$$

(11) $$\Phi \left(r_{1}\right)=\frac{1-\cos\left(2\pi r_{1}\right)}{1+\log\left(1+r_{1}\right)}$$

$\Psi \left(r_{2}\right)$ is the nonlinear shrinkage factor; $\Phi \left(r_{1}\right)$ is the chaotic perturbation factor; γ is the shrinkage strength parameter; λ is the attenuation factor; $X_{\mathrm{cbest}\;}^{t}$ denotes the current iteration optimal solution; $X_{\mathrm{best}\;}^{t}$ is the globally optimal solution; $tanh\left(\gamma r_{2}\right)$ maps random numbers $r_{2}\in [0,1]$ to smooth bounded ranges [0,tanh(γ)] to avoid oscillations due to large step sizes. γ controls the steepness of the curve, with larger values of $r_{2}$ being more sensitive to small changes. Exponential decay term $e^{-\lambda r_{2}^{2}}$: Suppresses the step size of large $r_{2}$-values, creating a “small step size for high frequency, large step size for low frequency” search pattern.

The $\Phi \left(r_{1}\right)$ generates a non-monotonic and asymmetric perturbation pattern within the interval $r_{1}\in (0,1)$, producing differentiated disturbance intensities at positions $r_{1}=0.25$ and 0.75. Compared with other chaotic mappings, this approach has lower computational cost and does not require iterative sequence generation. The use of two independent random variables (one governing the perturbation strength and the other controlling the search direction) forms a two-dimensional probabilistic space, which adds a broader range of search patterns than would be possible with a single random number. This design has proven effective in overcoming local optima in constrained search corridors.

### 3.2. Environment-Aware Boundary-Handling Strategy

In the original DBO algorithm, when an individual violates the search boundary, a simple truncation or directly setting the transboundary component to a boundary value is often used to ensure feasibility. There are two main problems with this approach: information loss: directly “trimming” individuals may destroy their original directional and positional information, especially in the critical region, which may lead to some valuable search information being ignored; and too much randomness: the lack of directional guidance when resetting to the boundary may destroy convergence.

Inspired by this, we designed an environment-aware boundary-handling strategy that treats the boundary as an “energy wall” and intelligently rebounds particles along the gradient field upon collision, using a three-phase process: collision detection (identifying the transgressed dimension), gradient sensing (calculating the gradient of the objective function at the boundary), and intelligent rebounding (adjusting the position along the direction of gradient descent). Specifically, as in Equation (12):

For individual $X_{i}$ in dimension d transgression:

(12) $$X_{i,d}^{\mathrm{new}}=\left\{\begin{array}{ll} {\mathrm{ub}}_{d}-\eta \left|X_{i,d}-{\mathrm{ub}}_{d}\right|\cdot \mathrm{sign}\left(\nabla_{d}f\right) & \;\mathrm{if}\;X_{i,d}>{\mathrm{ub}}_{d}\\ {\mathrm{lb}}_{d}+\eta \left|{\mathrm{lb}}_{d}-X_{i,d}\right|\cdot \mathrm{sign}\left(\nabla_{d}f\right) & \;\mathrm{if}\;X_{i,d}<{\mathrm{lb}}_{d} \end{array}\right.$$

The center difference method is used to avoid double counting:

(13) $$\nabla_{d}f\approx \frac{f\left(X+\epsilon e_{d}\right)-f\left(X-\epsilon e_{d}\right)}{2\epsilon }$$

where η: rebound factor; $\nabla_{d}f$: gradient direction in the d dimension; $e_{d}$: unit vector in the d dimension; ϵ: minor perturbation.

This environment-aware boundary-handling strategy significantly enhances the performance of the optimization algorithm on constrained problems through a gradient-guided intelligent rebound mechanism. On the one hand, by maintaining the directionality of the search, particles rebound along the direction of the gradient of the objective function when they cross the boundary, thus avoiding the loss of information caused by the traditional random reset, and prompting the search process to always be directed toward the favorable region; on the other hand, through the adaptive step-size adjustment, the fine search is conducted near the boundary in order to enhance the exploitation of the boundary region, effectively avoiding the premature convergence and at the same time maintaining the diversity of the populations.

### 3.3. Dynamic Attraction–Repulsion Force-Field Mutation Strategy

The original DBO algorithm relies only on attraction to the global optimal solution, which leads to rapid population aggregation and loss of diversity, thereby increasing the risk of premature convergence. Moreover, all dimensions are updated synchronously, which cannot deal with nonlinear correlation between variables, and the fixed or linear decay step mechanism is difficult to adapt to the demand of multi-stage optimization.

Based on this, a dynamic attraction–repulsion force field strategy is designed: the suboptimal solution with the top 10% fitness is selected as the source of repulsion so that the algorithm is able to perform a fine local search when it is close to the global optimization, and at the same time the repulsion force is utilized to prevent premature aggregation and maintain population diversity. The repulsive force strength is enhanced with iterations as specified in Equation (14):

(14) $$F_{i}=w_{g}\frac{X_{\mathrm{best}\;}-X_{i}}{{\left\|X_{\mathrm{best}\;}-X_{i}\right\|}^{3}}-w_{r}\sum_{j\in K}\frac{X_{j}-X_{i}}{{\left\|X_{j}-X_{i}\right\|}^{3}}$$

Intelligent mutation rules:

(15) $$X_{i}^{\mathrm{new}\;}=\left\{\begin{array}{ll} X_{i}+F_{i}\cdot \eta_{\mathrm{guided}\;} & \;\mathrm{if}\;\mathrm{rand}\;<p_{m}\\ X_{i}+N\left(0,\sigma_{t}^{2}\right) & \;\mathrm{else}\; \end{array}\right.$$

where $w_{g}=w_{0}\left(1-\frac{t}{T}\right)$ is the time-varying attractive weight; $w_{r}=\frac{t}{T}w_{0}$ is the time-varying repulsive weight; K is the set of suboptimal solutions in the top 10% of fitness; $\eta_{\mathrm{guided}\;}=1-e^{-\left\|F_{i}\right\|}$ is the nonlinear step size; $\sigma_{t}=\sigma_{0}\left(1-\frac{t}{T}\right)$ is the adaptive factors perturbation; this strategy is favorable in the weak-field region ($\left\|F\right\|\to 0$): fine search with small step sizes; and in the strong-field region ($\left\|F\right\|\gg 1$): fast moving with large step sizes. In the case of the multiple-peak function: the repulsive field effectively prevents premature convergence, and in the case of the single-peak function: the attractive field accelerates the convergence rate. Pseudo code such as the Algorithm 2.

The Pseudo-Code of ECFDBO:


**Algorithm 2: ECFDBO (Environment-aware Chaotic Force-field Dung Beetle Optimizer)**
Input: population size: N; problem dimension: d; search boundary: [lb, ub]; maximum number of iterations: T_max_; fitness function: f(x) Output: Optimal position: X_best_1:      Initialize {X_i_}₁ⁿ ∼ Uniform (lb, ub) 2:      Evaluate fi, set X_best_ 3:      for t = 1…T_max_ do 4:                  // Construct the set of suboptimal solutions Q 5:                  Sort {X_i_} by fitness, let Q ← best K = max (3, ⌊0.1·N⌋) 6:                  for each i = 1…N do 7:                        Compute 8:                                  F_i_ ← F_g_ − F_r_← Calculated using Equation (14) 9:                              if rand () < pm then 10:                                  η ←Calculated using Equation (15) 11:                                  X_i_ ←Attraction–Repulsion Mutation 12:                             else 13:                                    // Chaos Steps Update 14:                                    X_i_ ← Calculated using Equation (9) 15:                             end if 16:                             X_i_ ← SmartReflect(X_i_, lb, ub, ε) ← Calculated using Equation (12) 17:                 end for 18:                 {X_i_}← DBO_ Stage({X_i_})      // Rolling, Reproduction, Foraging, Stealing 19:                 Evaluate all f_i_, update X_best_ 20:    end for 21:    return X_best_

## 4. Complexity Analysis

The time complexity is an important index of the computational efficiency of the algorithm, assuming that the population size is N, the problem dimension is D, and the number of iterations is $T_{\max}$. The complexity of the original DBO algorithm is mainly influenced by the population initialization complexity (O(N⋅D)) and the complexity of the iteration process (O(N⋅D)), and the time complexity of the DBO algorithm is shown in Equation (16).

(16) $$O_{DBO}=O(N\cdot D)+T_{\max}\cdot O(N\cdot d)\approx O(T_{\max}\cdot N\cdot D)$$

ECFDBO adds three key strategies to the original DBO algorithm. The total complexity of its dynamic attraction–repulsion variation strategy: $O(N\cdot K\cdot D)=O(N^{2}\cdot D)$, where K≈0.1N are suboptimal solutions; the complexity of the individual behavior update: O(N⋅D); and the complexity of the environment-aware boundary processing strategy: $O(N\cdot D\cdot C_{f})$, where $C_{f}$ is the time complexity of a single adaptation. For each updated individual, it is necessary to check whether D variables are out of bounds and correct them, but usually the proportion of out-of-bounds individuals is limited and the actual complexity is far less than the upper bound, so the time complexity can be approximated as O(N⋅D). The ECFDBO time complexity is calculated as shown in Equation (17)

(17) $$O_{ECFDBO}=O(T_{\max}\times N\times K\times D)\approx O(T_{\max}\times N^{2}\times D)$$

From the analysis, it is evident that the complexity of ECFDBO increases from linear to quadratic growth of the original algorithm, primarily due to the attraction–repulsion field. Despite the increase in time complexity, this increase is acceptable under modern computing conditions. The introduced strategy reduces the probability of the algorithm falling into a local optimization and improves the overall convergence performance so that the increased time cost in exchange for a significant improvement in performance is reasonable and worthwhile in most practical optimization scenarios.

## 5. CEC2017 Test

The CEC series of test suites, recommended by the IEEE CEC Conference, has become a standard evaluation platform in swarm intelligence and evolutionary algorithms, ensuring fair and reproducible comparisons among different algorithms. In order to fully evaluate ECFDBO’s performance advantages, ECFDBO algorithms, and some classical and novel meta-heuristic algorithms are compared based on the CEC2017 test suite, which has wide recognition. All experiments of this research work were performed on Windows 10 64-bit, Intel(R) Core (TM) i5-12400 processor (2.50 GHz), 16 GB RAM, and MATLAB R2023a.

### 5.1. CEC2017 Introduction

The CEC2017 test suite aims to provide a fair and systematic performance evaluation of the newly proposed optimization algorithms. The test suite contains 29 standardized single-objective real-parameter optimization functions (the F2 function has been officially removed due to instability), including two single-peak functions (F1–F3) to test the global convergence capability of the algorithm; seven multi-peak functions (F4–F10) to test the global exploration capability of the algorithm; ten hybrid functions (F11–F20) to evaluate the performance of the algorithm by combining different basis functions; and 10 composition functions (F21–F30) to test the robustness and adaptability of the algorithm to highly complex and nonlinear problems. Both simple convex functions are covered, and high-dimensional complex combinations are introduced to ensure the reliable performance of the algorithm in diverse search spaces. The shift and rotation transformations are applied to all basis functions, and the search domain is uniformly set to: ${\left[-100,100\right]}^{d}$ to force the algorithm to find the optimal solution in a large range.

### 5.2. Comparison with Mainstream Optimization Algorithms

In order to facilitate the testing of the convergence capability, global exploitation capability, and robustness of the new improved algorithm, a total of seven comparison algorithms are compared and analyzed on the CEC2017 test component using five novel metaheuristic algorithms, namely BWO [37], COA [35], PIO [38], BOA [36], and NOA [39], as well as the original DBO [41] algorithm and a novel variant of DBO, namely GODBO [44]. Parameter settings are shown in Table 1. The test parameters are 30 populations, 500 iterations, and 30, 50, and 100 dimensions, and the results are evaluated on five performance indicators: mean, standard deviation, best, median, and worst. The optimal solution for each function is shown in bold.

**Figure A1:**
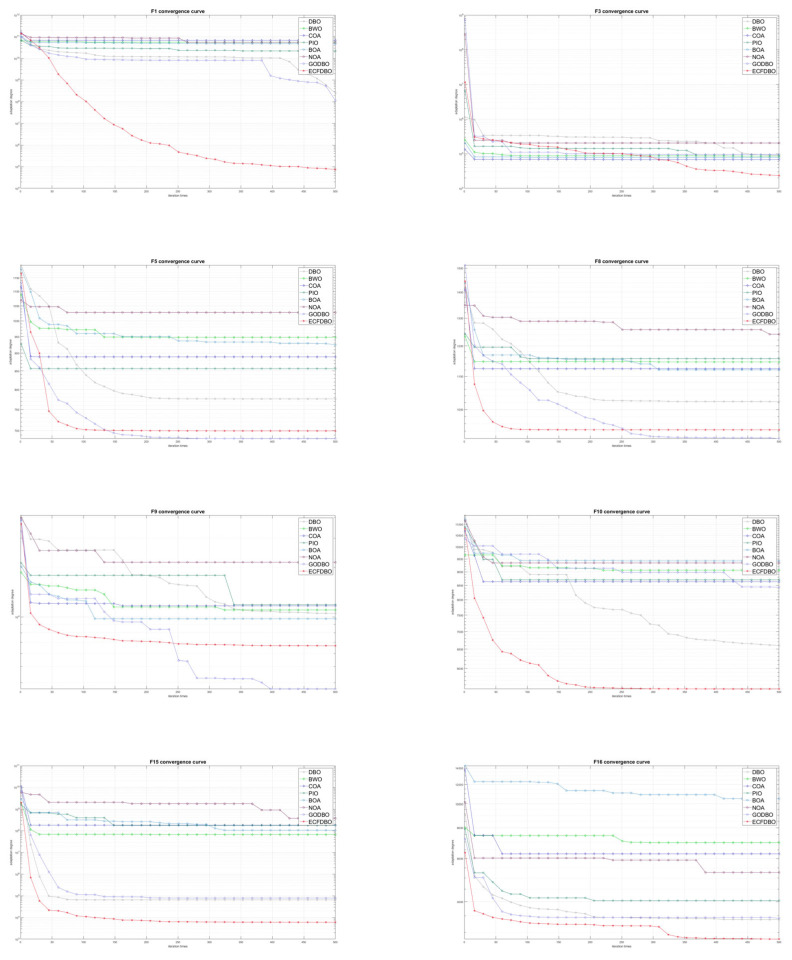

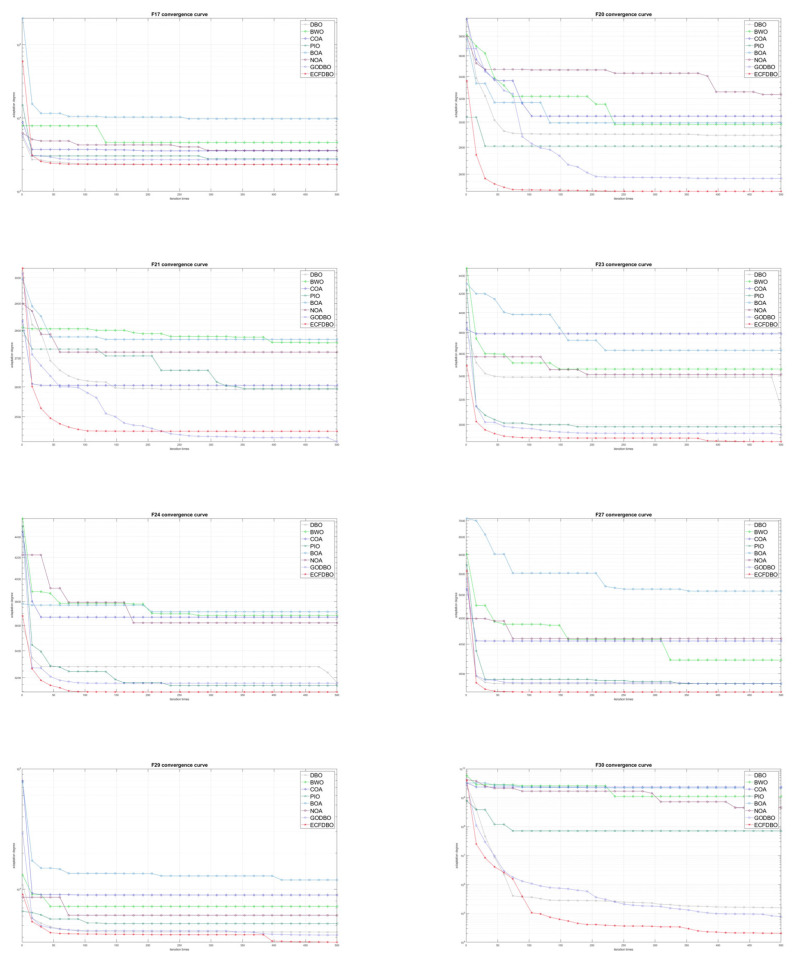
Convergence trends of classical functions in CEC 2017 (dim = 30).

As shown in Table 2 and Figure A1, in the 30-dimensional CEC2017 benchmark, the ECFDBO algorithm achieved the best average performance on most of the test functions. Among the 29 test functions, ECFDBO achieved the best performance on 18 functions, while GODBO performed best on the remaining 11 functions. It is worth noting that, with the exception of GODBO, none of the other algorithms compared can outperform ECFDBO on any function; for example, on the typical complex benchmark function F1, the average value of ECFDBO is only 63,942.51, while the suboptimal algorithm DBO is as high as 2.64 × 10^8^, and the rest of the algorithms are even in the order of 10^10^, which is an extremely wide gap, showing the significant advantage of ECFDBO in convergence precision. On simpler benchmarks such as the F5 function, although GODBO slightly outperforms ECFDBO (with means of 657.68 vs. 800.48), ECFDBO still significantly surpasses all other algorithms. In terms of standard deviation, ECFDBO achieved minimum values for 15 functions, followed by BWO with 7 functions. On the complex hybrid function F12, ECFDBO’s standard deviation is 1,863,468, which is 10^2^ better than the second best algorithm, DBO with 1.19 × 10^8^. On the F29 function, ECFDBO’s 271.7949 is only less than 50 different from the first place GODBO, and in the remaining optimal, median, and worst values, ECFDBO has 15, 19, and 17 optimal function values, respectively, all of which are ranked first among all algorithms. Overall, ECFDBO shows clear dominance on the 30-dimensional problem: six of the seven algorithms compared are outperformed by ECFDBO on almost all functions, and only GODBO is able to follow ECFDBO on some functions.

**Figure A2:**
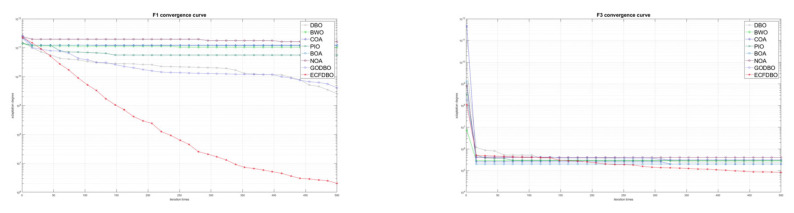

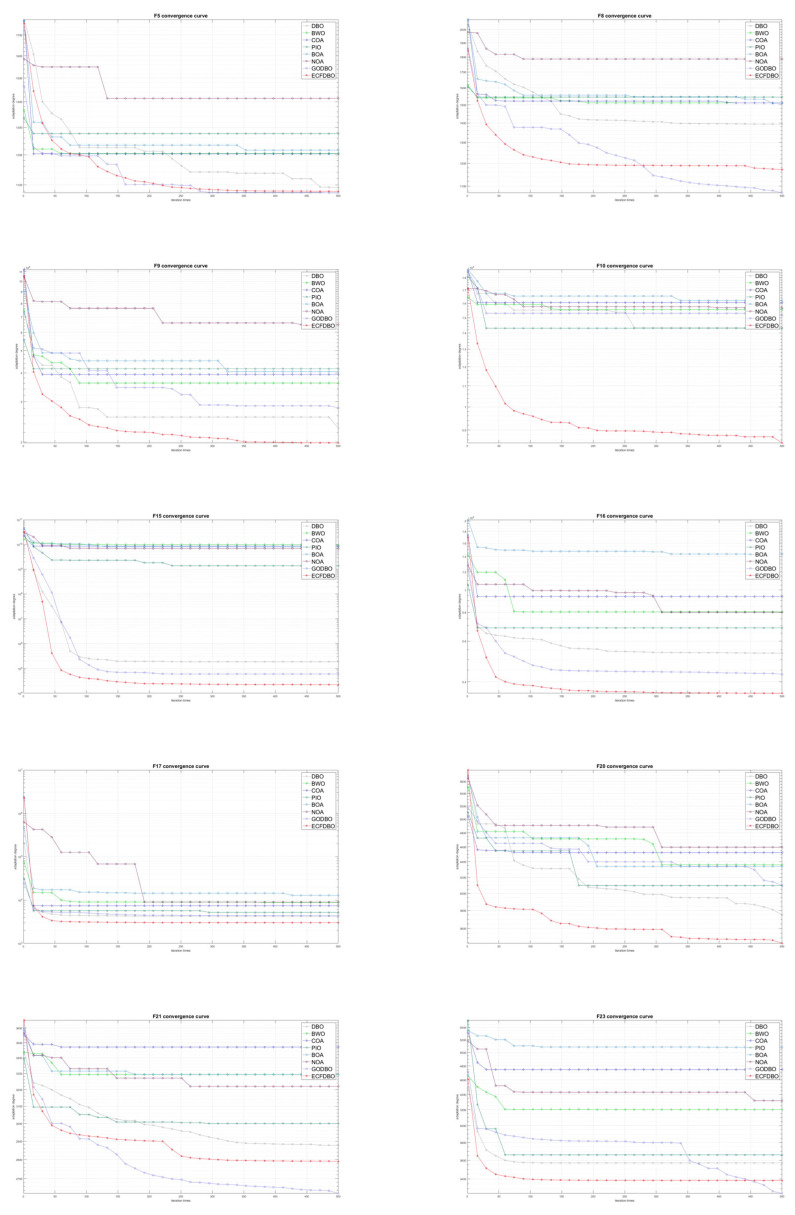

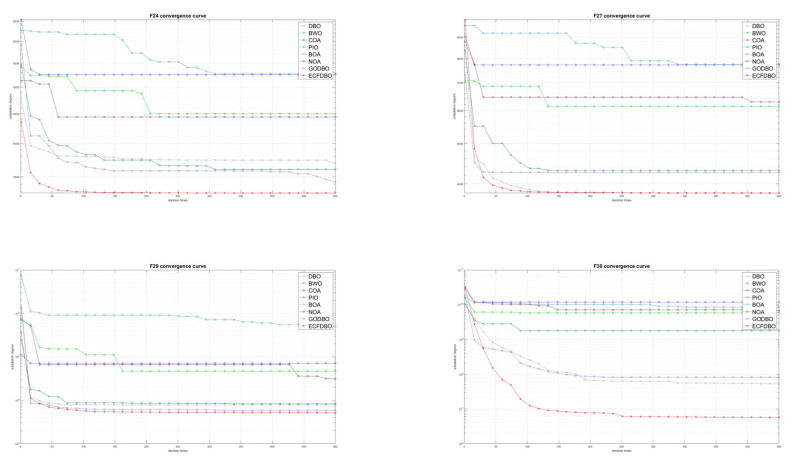
Convergence trends of classical functions in CEC 2017 (dim = 50).

As shown in Table 3 and Figure A2, the overall dominance of ECFDBO is further extended in the 50-dimensional benchmark test. The result statistics for the 29 tested functions show that ECFDBO achieves the best average performance value on 19 functions, while the remaining 10 functions are still dominated by GODBO. None of the other algorithms outperform ECFDBO on any function, reflecting the fact that ECFDBO remains a solid leader in higher dimensions. For example, on the F9 function, ECFDBO has a mean of 2.63 × 10^4^, while the second-best GODBO is about 1.87 × 10^4^ higher, and the remaining algorithms’ values are generally more than an order of magnitude higher. This indicates that as the dimensionality increases, some algorithms such as BWO and COA show deterioration in performance, with their average errors and rankings significantly lagging behind those of ECFDBO; in contrast, ECFDBO and GODBO are still able to maintain good adaptation to complex functions. In terms of standard deviation, ECFDBO achieves the lowest fluctuation on 14 functions, and the second-best BWO is the most stable on 5 functions; in the complex single-peak function F1, the standard deviation of ECFDBO is about 2.36 × 10^6^, while the value of GODBO is more than 7.03 × 10^8^, with a magnitude difference of more than 10^2^. In the rest of the best, median, and worst values, ECFDBO also achieves the first place with 18, 17, and 18, while GODBO is in the second place with 10, 9, and 10. In the high-dimensional environment of 50 dimensions, the ECFDBO algorithm still shows the overall leading performance advantage. Its average convergence precision continues to be the best and is slightly better than the performance in 30 dimensions. In the extreme cases of optimal/worst, ECFDBO wins with the majority function, showing good robustness.

**Figure A3:**
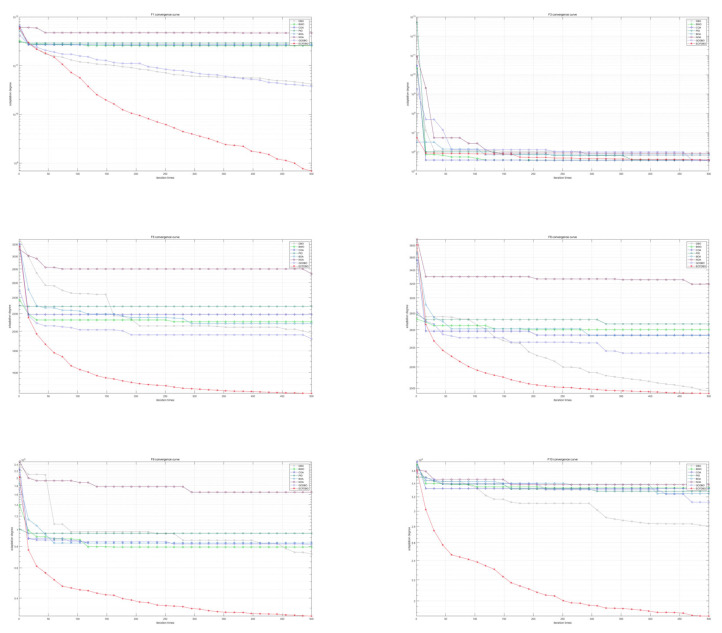

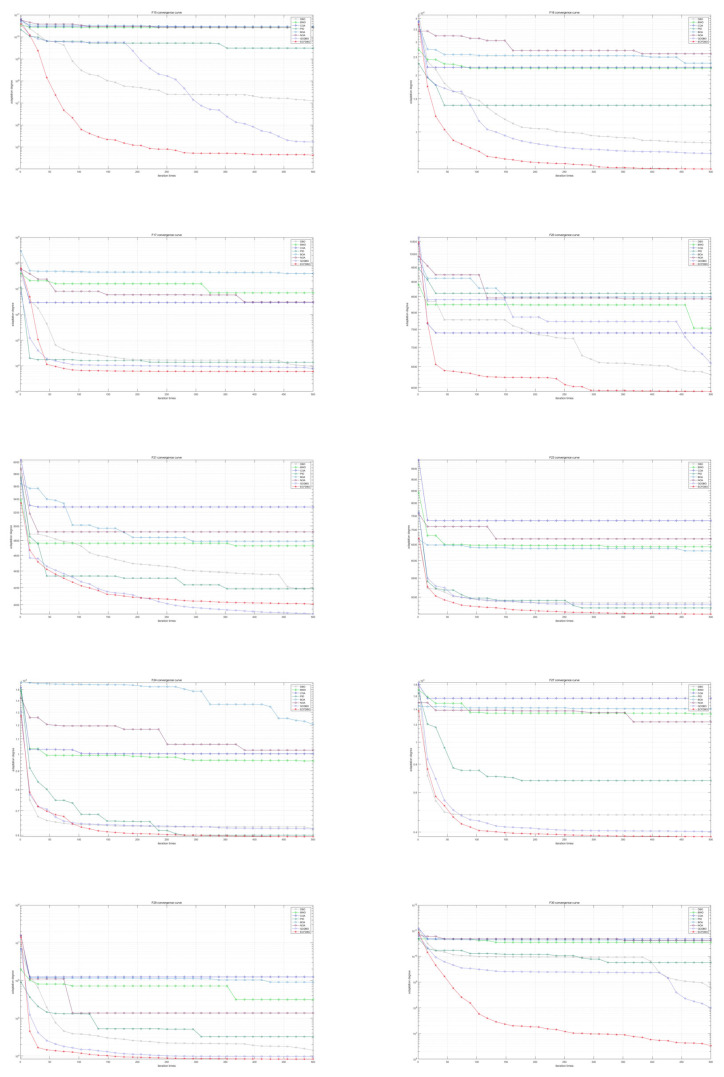
Convergence trends of classical functions in CEC 2017 (dim = 100).

As shown in Table 4 and Figure A3, in the 100-dimensional benchmarks, the performance of the algorithms is more clearly differentiated: ECFDBO remains the best on most functions, with the best average optimization on a total of 20 functions; GODBO is still the main contender, with a slight edge on 8 functions; and there is an unexpected optimization of the COA algorithm on F3, with an average of 3.51 × 10^5^ for COA and 5.56 × 10^5^ for ECFDBO, suggesting that ECFDBO may fall into a local optimization on this particular function. Nevertheless, ECFDBO still performs best on most functions. For example, on the F7 function, which has a complex multi-peak structure, ECFDBO averages 3.29 × 10^3^, significantly outperforming all algorithms except GODBO (2.75 × 10^3^); even on the F3 function, which has a dominant COA, ECFDBO ranks 4th without significant performance degradation. In terms of standard deviation, best, median, and worst, ECFDBO wins again with 14, 18, 17, and 18 times, respectively. Especially in terms of standard deviation, the suboptimal algorithm BWO has only 5 optimal times, GODBO is only dominated by one function, F29, and DBO has even failed completely, creating a substantial performance gap with ECFDBO. Overall, ECFDBO shows more stable and efficient optimization in 100 dimensions compared to 30 dimensions and 50 dimensions.

Combining the experimental results in 30, 50, and 100 dimensions, ECFDBO consistently leads in average performance, stability, and extreme cases, demonstrating good scalability and robustness as dimensionality increases, and it shows better overall solution quality and stability of results on the CEC2017 test set. As the dimensionality of the problem increases, the difference in solution accuracy between the algorithms widens significantly. ECFDBO achieving the lowest mean and std for most of the test functions in all dimensions, indicating that its converged solutions are closer to the global optimization with less variation. As the dimensions increase from 30 to 100, ECFDBO grows steadily from 18, 19 to 20 functions to reach the optimal mean. This shows that ECFDBO adapts well to dimensional extensions and maintains strong solving power on high-dimensional problems. In contrast, other algorithms such as BOA, BWO, PIO, etc. show a significant decrease in solution accuracy as dimensionality increases. This reflects that these algorithms are prone to local optimization or unstable convergence in high-dimensional complexity, while ECFDBO effectively alleviates the performance degradation caused by the increase in dimensionality through improved strategies.

### 5.3. Wilcoxon and Friedman Statistical Tests

In order to provide a more in-depth and systematic statistical analysis of the experimental results, the Wilcoxon and Friedman tests were introduced in this study to quantify the significance of the performance differences between the ECFDBO algorithm and the seven comparison algorithms. Specifically, the Wilcoxon test performs a test on the paired observations of ECFDBO and each comparison algorithm in the dataset, where the difference in performance between the two algorithms is considered statistically significant if the *p*-value obtained from the test is <0.05 (shown in bold) when the significance level is α=0.05; otherwise, the difference is not statistically significant. The Friedman test aggregates the rankings of all algorithms across the functions in the CEC2017 benchmark suite and computes their average ranks. This process evaluates whether the overall differences among multiple algorithms across multiple test functions are statistically significant, thereby providing a global, nonparametric statistical basis for algorithm comparisons.

Based on Table 5, Table 6 and Table 7, the Wilcoxon test shows that ECFDBO has a significant advantage over most of the algorithms. Overall, the differences between ECFDBO and the seven algorithms BWO, COA, PIO, BOA, NOA, GODBO, and DBO reached a significant level (*p* < 0.05) in the function-by-function comparison with each of the compared algorithms in 30 dimensions, which proves that the results of ECFDBO are significantly better than these algorithms. The three algorithms, BWO, COA, and NOA, all showed significant differences during the comparison process with ECFDBO one by one. PIO had only two functions (F23 and F24), and BOA had one function (F22). GODBO had four functions (F17, F18, F20, and F24) and did not show significant differences, while the original DBO showed advantages with ten functions. When the number of dimensions reaches 50, ECFDBO has more significant advantages, including the original DBO and GODBO, which were second to ECFDBO in the previous 30 dimensions, and the advantages of ECFDBO over the original DBO and GODBO are further extended in the 50-dimensional benchmark. The remaining algorithms have more significant differences with ECFDBO; for example, the *p*-value is close to 0 for BWO and COA, etc. Four algorithms even show significant differences with ECFDBO in all 29 functions, proving that ECFDBO has a clear lead in higher dimensions. The performance improvement of ECFDBO over all seven algorithms compared to 100 dimensions was significantly better than the other algorithms, with a total of 11 functions with *p*-values > 0.05. It is worth noting that although the GODBO algorithm achieved the best results on individual functions in the previous test, the overall gap between it and ECFDBO is still extremely obvious—ECFDBO achieved 20 wins against GODBO in 100 dimensions. It can be seen that as dimensionality increases, ECFDBO outperforms all comparators in the statistical sense.

As shown in Table 8, the Friedman test shows that the ECFDBO algorithm has a lower average rank than the other algorithms on all dimensions, and the bright spot is that GODBO and the original DBO are ranked second and third on all dimensions. From this, it can be seen that the superiority of ECFDBO is not due to randomness but has reliable statistical support. There are almost no algorithms that can match or exceed ECFDBO’s overall performance in any dimension. This means that the performance improvement of ECFDBO over every other algorithm is statistically significant, with advantages over BWO, COA, and NOA being particularly prominent, and a reliable lead over second-tier algorithms such as GODBO and DBO. Thus, it can be quantitatively confirmed that ECFDBO significantly outperforms the seven meta-heuristic algorithms compared to the CEC2017 test set as a whole, demonstrating robust optimization capabilities in all dimensions. For this statistical advantage, the excellent performance of the ECFDBO algorithm on each benchmark function fully demonstrates its effectiveness and competitiveness in solving complex optimization problems.

### 5.4. Contribution of the Improvement Strategies

ECFDBO brings three key improvement strategies for the DBO algorithm to enhance its performance.

Chaotic Perturbation Mixed Nonlinear Contraction Mechanisms: ECFDBO introduces chaotic sequence perturbation into the iterative process and adopts a nonlinearly decreasing convergence factor to regulate the search step size so as to dynamically balance global exploration and local exploitation. Chaotic sequences can effectively increase the population diversity and improve the global search capability of the algorithm due to their ergodicity and randomness. In the early stage of the algorithm, the chaotic perturbation helps ECFDBO to explore the solution space more extensively to avoid falling into the local optimization prematurely, and as the iteration proceeds, the perturbation amplitude gradually shrinks according to the nonlinear function, which helps the algorithm to perform a fine search near the optimal solution at a later stage, thus improving the convergence precision. This adaptive perturbation mechanism makes up for the lack of perturbation means in the original algorithm: lack of population perturbation, the algorithm can easily fall into the local optimization and missing the opportunity to search other regions. Therefore, chaotic perturbation combined with nonlinear contraction strengthens the global exploration capability and late convergence stability of ECFDBO in complex multi-peak environments, dramatically reducing the solution deviation and improving the robustness of the results.

Environment-Aware Boundary-Handling Strategy: For the problem of candidate solutions crossing the boundary, ECFDBO adopts an environment-aware boundary-handling mechanism. When an individual crosses the boundary of the defined area, the algorithm does not simply cut it back to the boundary or reset the random value but adjusts it according to the environmental information at the time the individual crosses the boundary so as to maintain the feasibility of the solution without losing diversity. Proper boundary handling is critical to the performance of population intelligence algorithms, and ECFDBO’s boundary handling takes into account the fitness environment of the individual’s region, intelligently pulling out-of-bounds solutions back into potentially favorable regions of the search space rather than blindly discarding or zeroing them. This ‘environment-aware’ approach improves the efficiency of the algorithm’s search near the boundary, avoiding the traditional hard boundary handling that can cause individuals to oscillate or stagnate at the boundary.

Dynamic Attraction–Repulsion Force-Field Mutation Strategy: ECFDBO introduces a mutation operator that simulates the effects of attractive and repulsive forces to speed up convergence while maintaining population diversity. The strategy applies an ‘attractive force’ to the high-quality solutions to bring the surrounding individuals closer to them so as to use the global optimal information to guide the search and improve the speed of local convergence; at the same time, it applies a ‘repulsive force’ to the over-aggregated individuals to push them away from their current position to encourage the population to spread to unexplored areas and maintain the global search capability. Through Attractive -repulsive mutation, the population of ECFDBO can consistently jump out of local traps while approaching the global optimization. It makes the algorithm rarely stagnate in local extremum traps of complex functions and speeds up the optimality search process of the global optimization.

In the relationship between the 3D path planning task of unmanned aerial vehicles (UAVs) and the CEC2017 test experiments: In terms of the correspondence of problem characteristics, ECFDBO significantly outperforms the comparative algorithms in the 30D/50D/100D tests of CEC2017, demonstrating its remarkable high-dimensional optimization capabilities. Given that UAV path planning involves optimizing dozens of variables simultaneously in a 3D space, such as path length, threat avoidance, and altitude constraints, the high-dimensional optimization capabilities of ECFDBO can effectively address such problems. The excellent performance of ECFDBO in the multi-peak functions (F4-F10) and hybrid functions (F11-F20) of CEC2017 enables it to help UAVs find a globally safe path amidst the “traps” of local optimal solutions formed by multiple obstacles. The intelligent boundary-handling mechanism of ECFDBO, as evidenced by its performance in the constrained functions of CEC2017, ensures that UAVs strictly comply with physical constraints such as the flight altitude range and minimum path length. Regarding the problem goals, the advantages of ECFDBO in terms of convergence speed and results in CEC2017 guarantee that UAVs can quickly find the optimal solution during path planning. The comprehensive advantages of ECFDBO in the composite functions (F21-F30) can assist UAVs in generating optimal paths under conditions of multiple conflicting constraints.

## 6. Collaborative 3D Path Planning Simulation

Path planning in complex spatial environments is a critical step for UAV navigation in remote sensing missions. It ensures that the UAV safely and efficiently travels from one location to another while satisfying all mission-related constraints and requirements [51]. Based on these considerations, the model developed in this study is constructed as follows.

### 6.1. Problem Statement

#### 6.1.1. Flight Path Distance

In order to make the operation of UAVs more relevant to actual needs, the path planning should be based on different application scenarios for goal setting and constraints. Since we focus on remote sensing and surface monitoring, etc., we assume that there are a total of N UAVs, and the path of each UAV i consists of a series of discrete points, and the flight path of the UAVs is represented as a list consisting of a number of discrete points, $P_{i}=\{p_{i,1},p_{i,2},\ldots ,p_{i,m_{i}}\}$: the set of path points of the UAVi, where $p_{i,k}=(x_{i,k},\;y_{i,k},\;z_{i,k})$; the total flight path length of the flight path of the UAVi is represented as Equation (18):

(18) $$L_{i}=\sum_{k=1}^{m_{i}-1}\left\|p_{i,k+1}-p_{i,k}\right\|$$

Let the sum of the theoretical shortest path lengths of all UAV origins and destinations be $L_{\max}$, where the path cost from the origin to the destination is expressed as Equation (19):

(19) $$f_{o}=p_{1}\cdot \frac{\sum_{i=1}^{N}L_{i}}{L_{\max}}$$

where  p1 is the adjustment factor. By minimizing $f_{0}$, it is possible to minimize the total range of the planned path, thus increasing the efficiency of the flight.

#### 6.1.2. Security and Threat Constraints

**Figure 2:**
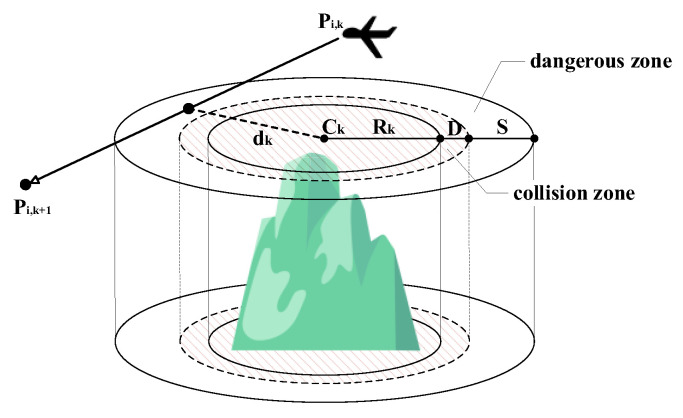
Cylinder threat.

In complex environments, UAVs must avoid static obstacles or airborne threats to ensure flight safety, where safety constraints are defined as cylindrical and spherical zones. Assuming there are K obstacles in the environment, each obstacle is represented by a cylindrical zone with horizontal projected circular center coordinates of $C_{k}$ and radius of $R_{k}$ (k=1,2,…,K). The safety radius of the UAV itself is denoted as D, and the safety distance to be maintained outside the collision zone of the UAV and the obstacle is defined as S. Let the closest distance to the center of an obstacle when the flight segment $\left\|\vec{P_{i,k}P_{i,k+1}}\right\|$ passes through the projection area of the obstacle be $d_{k}$ (e.g., Figure 2). Based on the distance of the UAV relative to the obstacle, the threat cost function $T_{k}$ of the flight segment relative to the obstacle k can be defined as in Equation (20) below:

(20) $$T_{k}(\vec{P_{i,k}P_{i,k+1}})=\left\{\begin{array}{ll} 0, & \mathrm{if}\;d_{k}>S+D+R_{k}\\ \frac{(S+D+R_{k})-d_{k}}{S}, & \mathrm{if}\;D+R_{k}<d_{k}\leq S+D+R_{k}\\ 1, & \mathrm{if}\;d_{k}\leq D+R_{k}. \end{array}\right.$$

where

(1) If $d_{k}\leq D+R_{k}$, the UAV has entered the collision zone of the obstacle, then the cost is taken as $T_{k}=1$ to indicate a collision;

(2) If $d_{k}>S+D+R_{k}$, the UAV is far enough away from the obstacle, take $T_{k}=0$;

(3) If $D+R_{k}<d_{k}\leq S+D+R_{k}$, the UAV has not collided but has entered the dangerous zone around the obstacle, and a penalty is applied to the path. The cost in this case is inversely proportional to the deviation from the distance and can be set to $T_{k}(\vec{P_{i,k}P_{i,k+1}})=\frac{(S+D+R_{k})-d_{k}}{S}$.

In summary, the total threat cost of the entire flight path relative to all obstacles can be cumulatively added to the threat surrogate value of each flight segment under each obstacle as in Equation (21):

(21) $$f_{no}=\sum_{j=1}^{n-1}\sum_{k=1}^{K}T_{k}(\vec{P_{i,k}P_{i,k+1}})\;.$$

For $UAV_{i}$ at waypoint $p_{i,k}$, if detected inside the  j-th threat sphere, distance $d_{i,k}^{j}$ is calculated; For spherical region 1, given a constant p31, the cost contribution can be defined as $c_{i,k}^{j}=\frac{p_{31}}{{\left(d_{i,k}^{j}\right)}^{4}}$; For spherical region 2, the following equation is used, assuming that spherical region 2 has a radius of $R_{a,j}$: $c_{i,k}^{j}=\frac{{\left(R_{a,j}\right)}^{4}}{{\left(R_{a,j}\right)}^{4}+{\left(d_{i,k}^{j}\right)}^{4}}.$ and that the cumulative traversal cost per UAV in all threat regions is $f_{t,i}=\sum_{k=1}^{m_{i}}\sum_{j\in T_{i}(k)}c_{i,k}^{j},$, where $T_{i}(k)$ denotes $UAV_{i}$ the set of threat regions detected at point k. The total threat traversal cost is then given by Equation (22):

(22) $$f_{t}=\sum_{i=1}^{N}f_{t,i}$$

With the threat-cost model described above, the cost will increase if the UAV’s path is too close to an obstacle, so the path planning will be directed away from the obstacle, and safety constraints will be met.

#### 6.1.3. Cost of Flight Altitude

**Figure 3:**
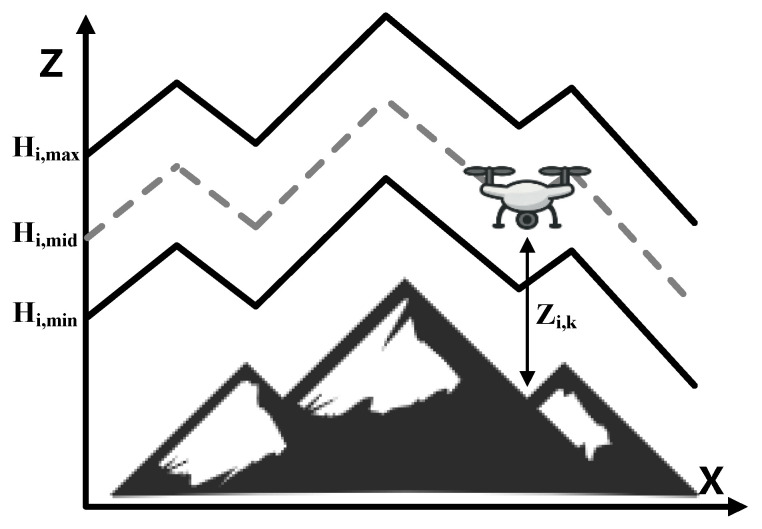
Cost of flight altitude.

In practice, in complex environments, the flight altitude of a UAV usually needs to be limited to a certain altitude based on the accuracy requirements of sensors or remote sensing equipment such as detection radar. The mission requirements (e.g., clarity of aerial imagery, sensor accuracy) will specify a minimum altitude $H_{i,min}$ and a maximum altitude $H_{i,max}$ as the lower and upper limits of the safe flight altitude for the UAVi and make the median safe value $H_{i,\mathrm{mid}}=\frac{H_{i,\min}+H_{i,\max}}{2}$. e.g., Figure 3.

The altitude constraint is therefore included in the cost function of the path planning. For the altitude $z_{i,k}$ of $UAV_{i}$ at each of its track points $\;p_{i,k}$, let the penalty function $h_{i\left(k\right)}$ be defined as Equation (23):

(23) $$h_{i}(k)=\left\{\begin{array}{ll} \frac{\left|z_{i,k}-Hi,mid\right|}{Hi,mid}, & \mathrm{if}\;H_{i,min}\leq z_{i,k}\leq H_{i,max}\\ 1, & \mathrm{otherwise}, \end{array}\right.$$

That is, if the waypoint altitude is within the permissible range $\left[H_{i,min},H_{i,max}\right]$, the cost is taken as the deviation from the median of the range to encourage the UAV to fly in the middle of the permissible altitude range, and the altitude cost of the path can be obtained by summing the altitude costs of the path for all the waypoints along the path, then the altitude cost model of the flight is given by Equation (24):

(24) $$f_{h}=\sum_{i=1}^{N}\sum_{k=1}^{m_{i}}h_{i}(k).$$

Modelling in this way ensures that the planned paths strictly adhere to the upper and lower altitude limits specified by the mission, with appropriate penalties for approaching the altitude limits, thus improving the mission fitness of the paths.

#### 6.1.4. Path Smoothness

**Figure 4:**
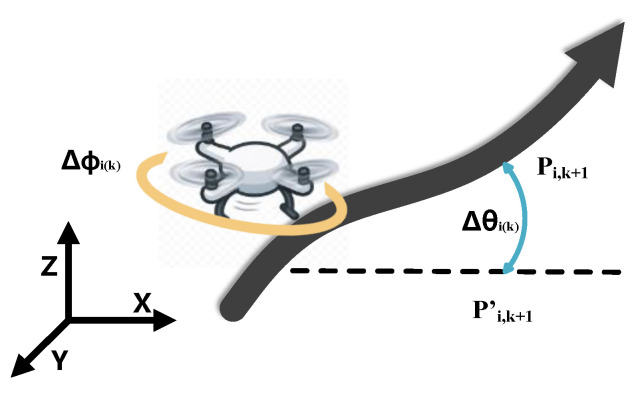
Path Smoothness.

To ensure a smooth UAV path, UAVs need to avoid violent steering and climb/dive maneuvers as much as possible. For this, two metrics, horizontal steering angle and climb angle, are introduced to quantify path smoothness, and their effects are incorporated into the cost function, e.g., Figure 4.

Firstly, the horizontal steering angle $\Delta \phi_{i(k)}$ is defined as the degree of turning of the path in the horizontal plane: for the three consecutive waypoints $P_{i,k}$, $P_{i,k+1}$ and $P_{i,k+2}$ in the path, their projection points on the horizontal plane XOY are ${P^{\prime }}_{i,k}$, ${P^{\prime }}_{i,k+1}$ and ${P^{\prime }}_{i,k+2}$, respectively, then the horizontal steering angle $\Delta \phi_{i(k)}$ can be defined as the angle between the projected segments $\vec{{P^{\prime }}_{i,k}{P^{\prime }}_{i,k+1}}$ and $\vec{{P^{\prime }}_{i,k+1}{P^{\prime }}_{i,k+2}}$, i.e., the magnitude of the horizontal steering angle, which is calculated using Equation (25):

(25) $$\Delta \phi_{i(k)}=\arctan\left(\frac{\left\|\vec{P_{i,k}^{\prime }P_{i,k+1}^{\prime }}\times \vec{P_{i,k+1}^{\prime }P_{i,k+2}^{\prime }}\right\|}{\vec{P_{i,k}^{\prime }P_{i,k+1}^{\prime }}.\vec{P_{i,k+1}^{\prime }P_{i,k+2}^{\prime }}}\right).$$

Secondly, the angle of climb $\Delta \theta_{i(k)}$ is used to describe the degree of inclination of the path in the vertical direction and can be defined as the angle of climb or descent of the segment $\vec{P_{i,k}P_{i,k+1}}$ with respect to the horizontal plane. It is usually calculated using Equation (26):

(26) $$\Delta \theta_{i(k)}=\arctan\left(\frac{\Delta z_{k}}{\left\|\vec{P_{i,k}^{\prime }P_{i,k+1}^{\prime }}\right\|}\right)$$

where $\Delta z_{k}=z_{i,k+1}-z_{i,k}$ is the height difference between adjacent waypoints and $\left\|\vec{P_{i,k}^{\prime }P_{i,k+1}^{\prime }}\right\|$ is the projected length of the segment in the horizontal plane. On the basis of these two angles, the change in declination and elevation angle is determined for each $UAV_{i}$ between its successive waypoints, defining the indicator function as in Equation (27):

(27) $$a_{i}(k)=\left\{\begin{array}{cc} 1, & \mathrm{if}\left|\Delta \phi_{i}(k)\right|>\phi_{\max}or\left|\Delta \theta_{i}(k)\right|>\theta_{\max},\\ 0, & \mathrm{otherwise}, \end{array}\right.$$

(28) $$A_{i}=\left\{\begin{array}{cc} 1, & \mathrm{if}\;\exists k\mathrm{make}s\;a_{i}(k)=1,\\ 0, & otherwise, \end{array}\right.$$

(29) $$f_{a}=\sum_{i=1}^{N}A_{i}.$$

where $\Delta \phi_{i(k)}$ and $\Delta \theta_{i(k)}$ are the change in declination and pitch angle, respectively, for segment k. Equation (28) is the angular constraint cost. By working together to form a cost function as in Equation (29), these two parts of the cost ensure that the generated path is smooth enough in 3D space to satisfy the UAV’s maneuverability constraints.

#### 6.1.5. Time Synchronization Constraint

For $UAV_{i}$, the total duration of its voyage is $t_{i}=\frac{L_{i}}{v_{i}}$, where $v_{i}$ is the average speed of the UAV. Then the time synchronization cost is defined by Equation (30):

(30) $$f_{m}=\sum_{i=1}^{N}p_{4}\cdot |t_{i}-t_{c}|$$

where p4 is the time synchronization weighting factor and the target synergy time is $t_{c}$.

#### 6.1.6. Space Collision Costs

**Figure 5:**
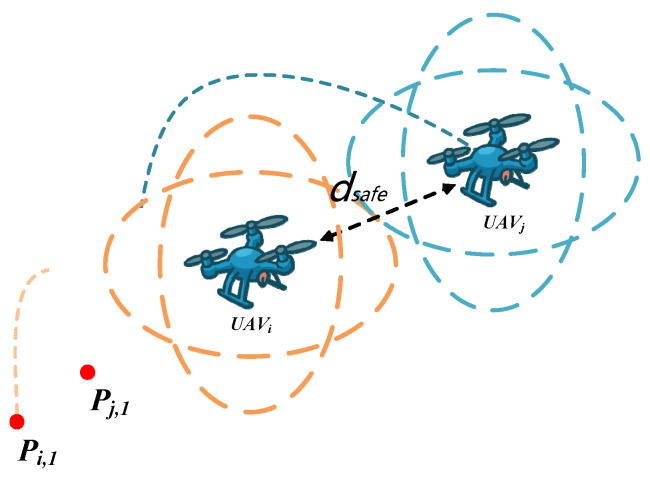
Safe distance for drones coordination.

As in Figure 5, assuming collision detection between different UAVs, the number of times that the distance between $\;UAV_{i}$ and $UAV_{j}$ is detected to be less than the safe distance $d_{safe}$ at the same time or time period is denoted as $C_{ij}$, where p5 is the spatial synergy or collision weight factor. The spatial synergy cost model can be written as Equation (31):

(31) $$f_{c}=p_{5}\cdot \sum_{1\leq i<j\leq N}C_{ij}$$

#### 6.1.7. Minimum Path Segment Interval Constraint

For each flight distance $\;l_{i,k}=∥p_{i,k+1}-p_{i,k}∥$ of $\;UAV_{i}$, when $l_{i,k}<L_{\min}$, $L_{\min}$ is a predetermined minimum allowable path segment length, the indicator function and the cost function can be defined as Equations (32) and (33):

(32) $$t_{i}(k)=\left\{\begin{array}{cc} 1, & \mathrm{if}\;l_{i,k}<L_{\min},\\ 0, & otherwise. \end{array}\right.$$

(33) $$f_{tr}=\sum_{i=1}^{N}\sum_{k=1}^{m_{i}-1}t_{i}(k)$$

Taking into account the path distance, obstacle threat, safety altitude range, and path smoothness, a comprehensive cost function (Equation (34)) for path planning can be established:

(34) $$F=w_{1}f_{o}+w_{2}f_{h}+w_{3}f_{t}+w_{4}f_{m}+w_{5}f_{c}+w_{6}f_{a}+w_{7}f_{tr}+w_{8}f_{no}$$

where $w_{1}$, $w_{2}$, $w_{3}$, …, $w_{8}$ are the weighting parameters of each cost component, which can be set according to the mission requirements and unit scale differences. Up to this point, the UAV cooperative 3D path planning problem is transformed into an optimization problem with a comprehensive cost function F as the objective.

Restrictions:

Waypoints $p_{i,1}$ and $p_{i,m_{i}}$ are fixed to the given start and end points;

For each waypoint $p_{i,k}$, $\;p_{i,k}\in \Omega$ must be satisfied, where Ω is the area in which the UAV can fly.

### 6.2. Simulation Experiment

**Figure 6:**
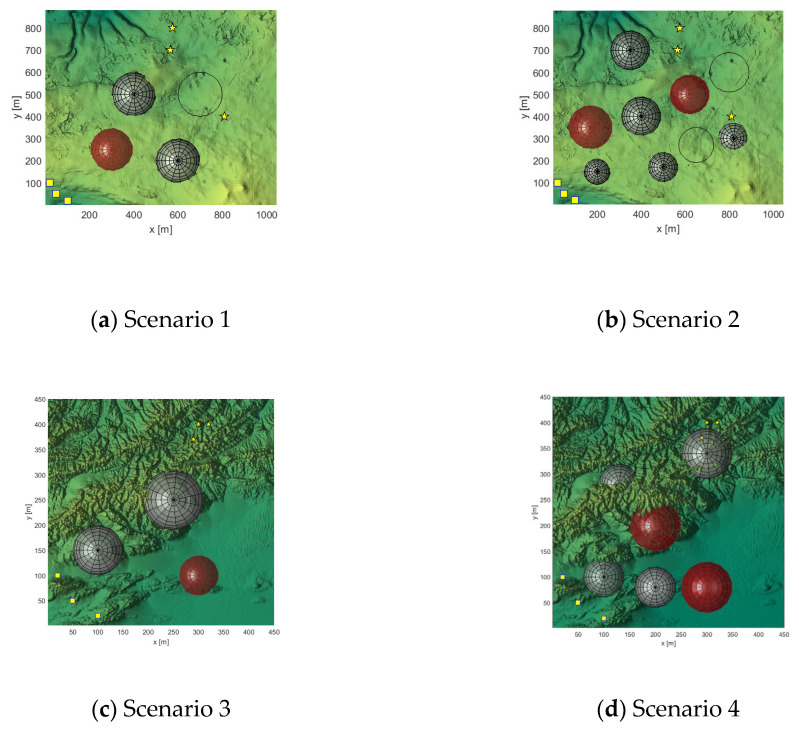
Layout of the four simulated scenarios.

In this experiment, two terrain maps and two threat layouts are set up, four application scenarios are simulated and validated, the UAV formation consists of three UAVs, and ECFDBO is used to compare the GODBO and the original DBO algorithms that performed well in the test. The experimental settings are population size N = 30, iteration number D = 200, and fitness function weights 0.05, 0.05, 2, 0.7, 0.7, 0.6, 0.9, 0.9, etc. In this experimental phase, the aim is to plan a safe path; therefore, safety-related costs such as security and threat constraints, collisions, and time synchronization are given high weights to ensure the safety and coordination of the task. In practical application, design can be carried out according to specific needs. The path points are set to 15 × 3. The layout of the experimental scenarios is shown in Figure 6.

We applied the eight algorithms to the four scenarios in Figure 6 for experimental validation, and each scenario was run independently 10 times, and the MEAN and BEST metrics of each algorithm were counted separately. From Table 9, we can see that in Scenario 1, ECFDBO has an average score of 1.5688, which is just behind COA (1.4832) and BWO (1.5763), but its best score (0.7681) is much better than all the algorithms except GODBO (0.3057). This shows that in simple environments, ECFDBO can not only maintain a more stable global search level but also better explore the optimal solution space. As the complexity of Scenario 2 increases, the average fitness of most algorithms increases. At this time, ECFDBO’s average fitness of 1.5242 remains low, and its optimal fitness reaches 0.1660, which is the lowest among all algorithms. In Scenario 3, ECFDBO’s best = 0.8294 is in first place. Scenario 4 further increases the search difficulty, and the mean and optimal values of most algorithms are significantly higher than those in Scenario 3. ECFDBO’s mean = 1.4131 and best = 0.8181 are still robust, while GODBO (mean = 2.9202, best = 2.1725) and the other algorithms fluctuate more in comparison. The optimal values are bolded in the table.

**Figure 7:**
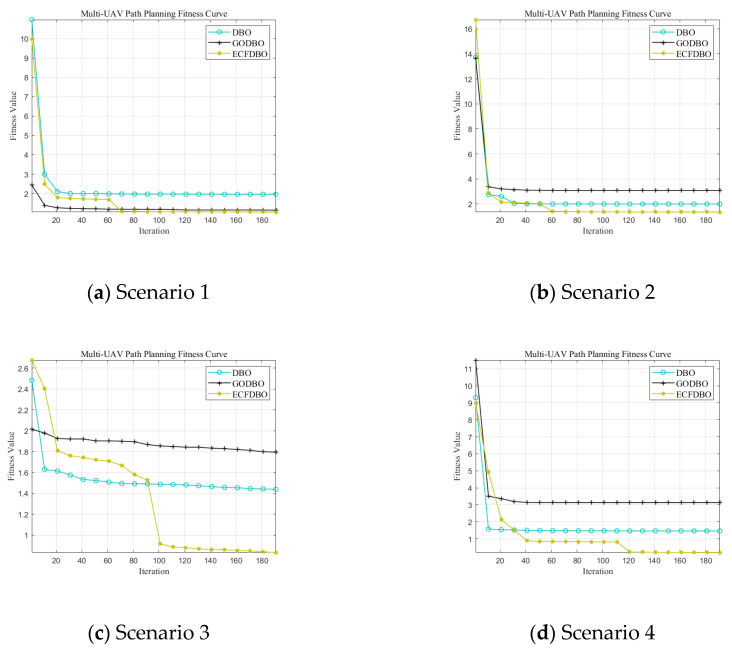
Comparison of fitness curves.

**Figure 8:**
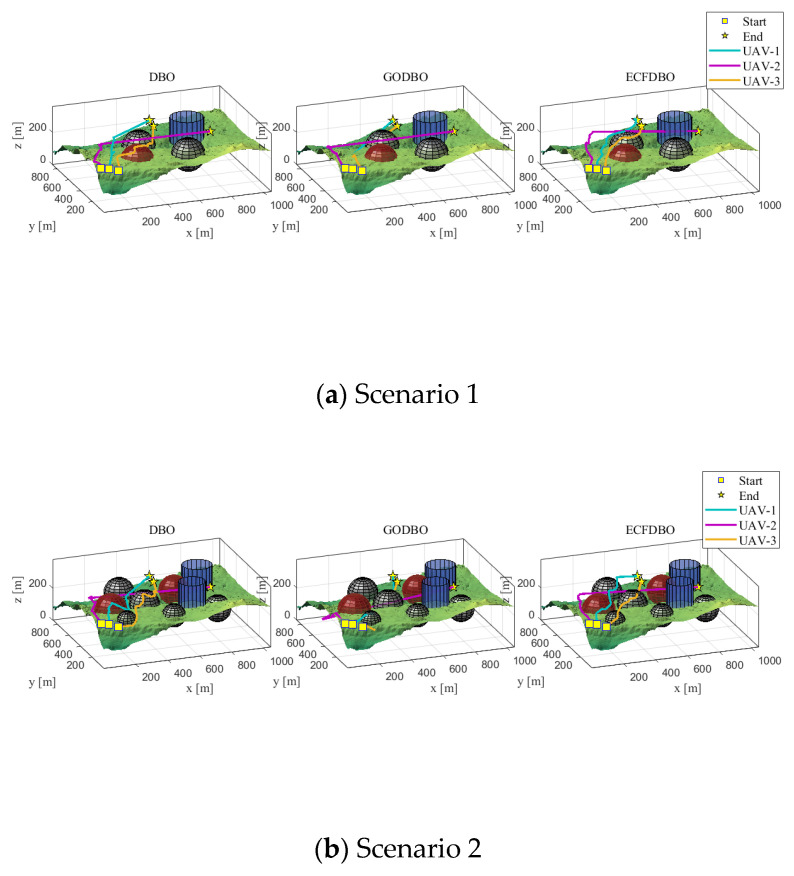

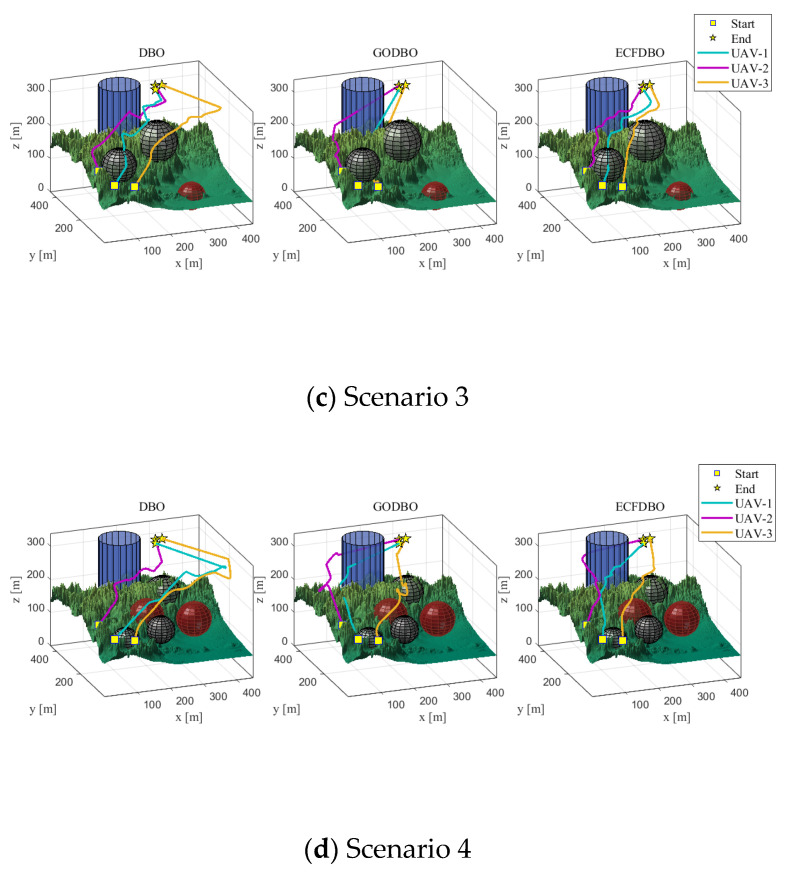
3D display of path.

**Figure 9:**
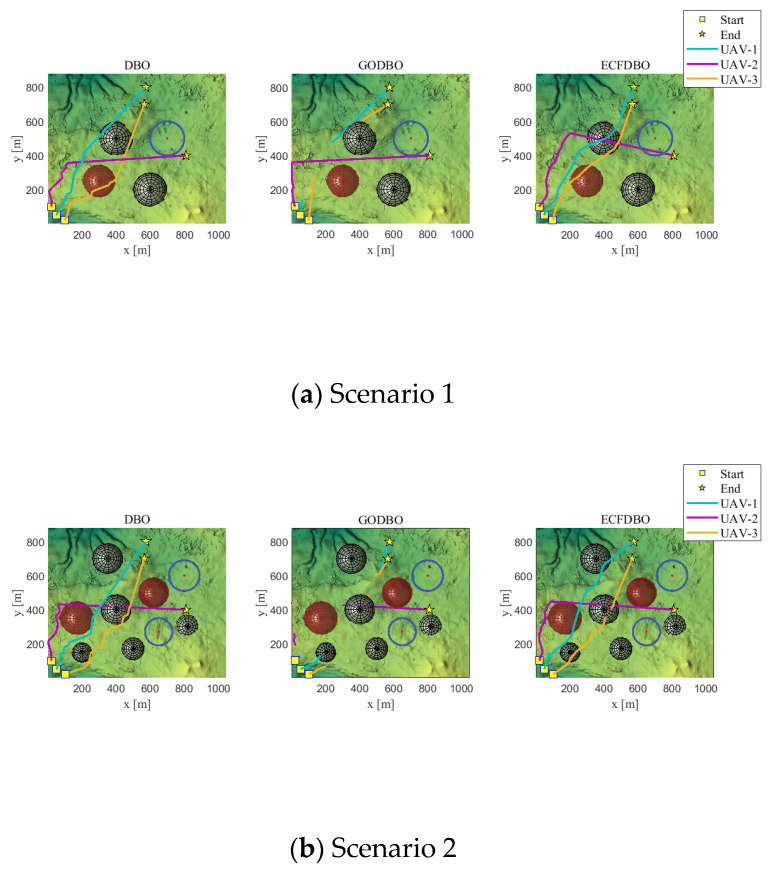

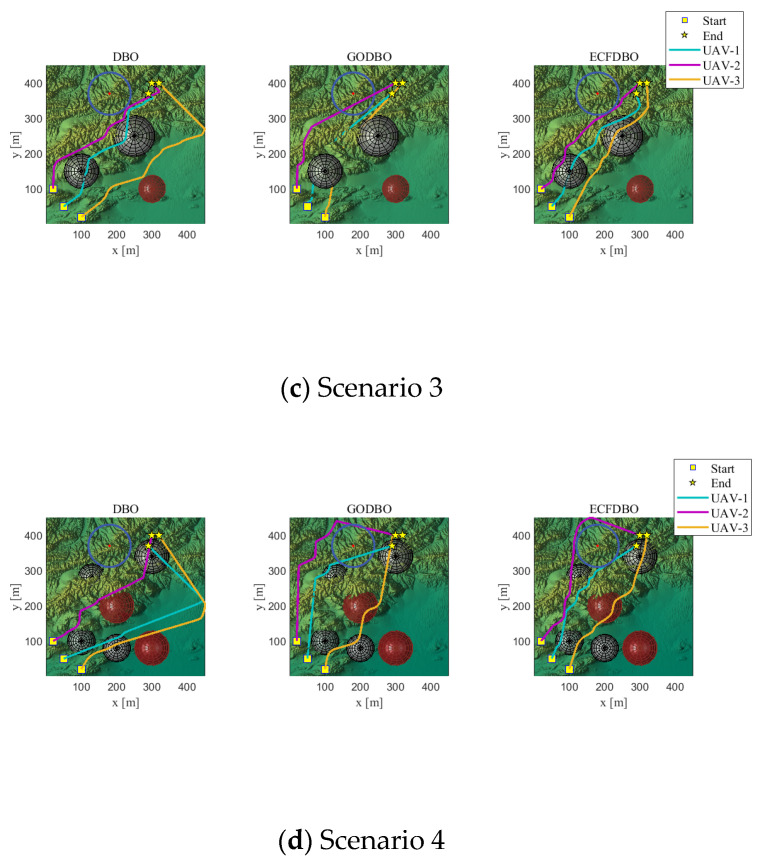
Vertical display of path.

**Figure 10:**
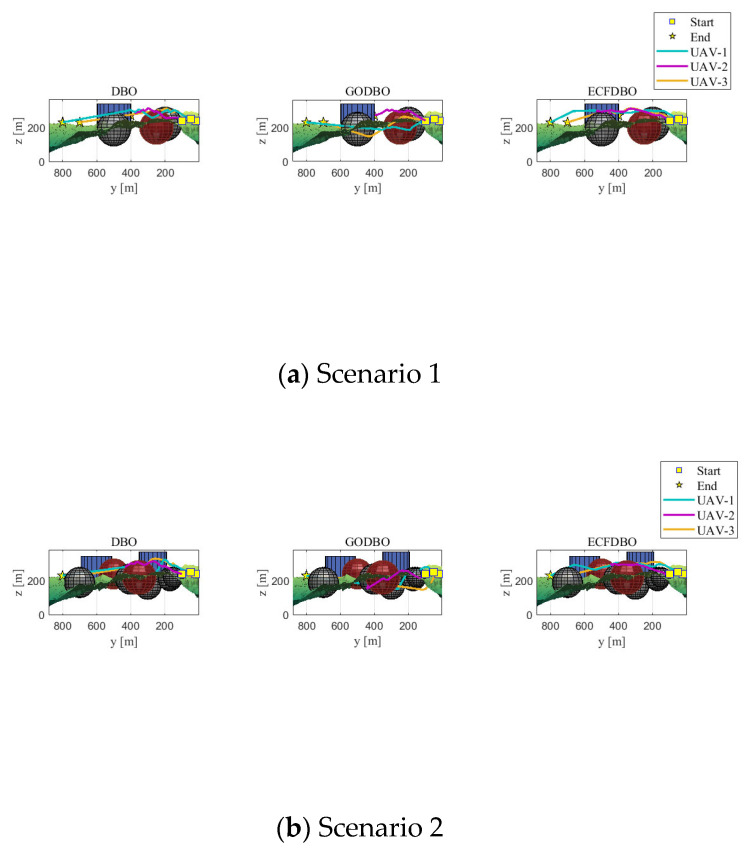

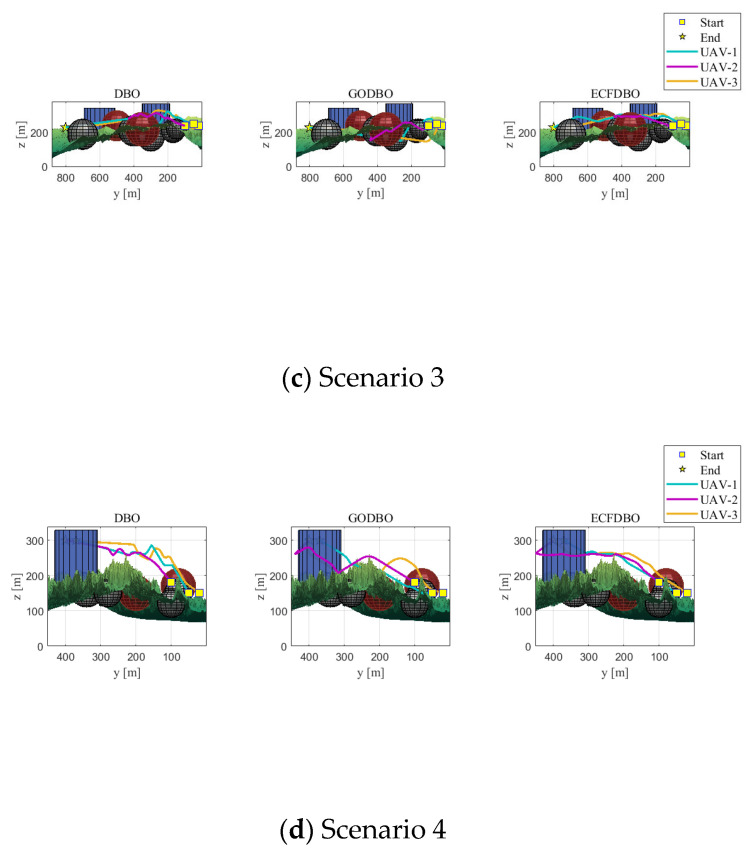
Height profile display of path.

The fitness iteration curves are shown in Figure 7. Scenario 1 terrain is relatively simple; obstacles are scarce and scattered; each algorithm can easily find a safe and feasible path. In terms of the convergence curve (Figure 7a), ECFDBO and GODBO converge to the lowest track cost during the early iterations and tend to be stable and optimal in about 40–70 generations; the final path cost of the original DBO is slightly higher than that of the former two. This shows that in a simple environment, each algorithm can find a better solution. Regarding path planning results, the three-dimensional paths (Figure 8a) and top view (Figure 9a) show that all three algorithms generally share similar paths and effectively avoid obstacles. ECFDBO has fewer inflection points and good smoothness; DBO’s planned path, while safe and feasible, is slightly stiff at some turns and has more track corners than ECFDBO. This is because the global search pressure of each algorithm in simple scenarios is not large; DBO can find feasible paths, but its local optimization ability is slightly inferior, and the path smoothness is somewhat poor. From the height profile (Figure 10a), it is evident that ECFDBO provides the best optimal altitude control. In general, ECFDBO finds the optimal path in Scenario 1, which verifies the algorithm’s effectiveness in simple environments.

Scenario 2 increases the number of safety constraints compared with Scenario 1, thus increasing the difficulty of path planning. From the convergence performance (Figure 7b), ECFDBO converges rapidly to the minimum cost in about 60 generations, showing excellent global optimization speed. DBO and GODBO converge in about 20–40 generations, but their minimum costs are slightly higher than that of ECFDBO. This indicates that the search accuracy of ECFDBO is significantly better than that of the other algorithms in this scenario. The path comparisons (Figure 8b and Figure 9b) reveal that all three algorithms exhibit varying degrees of constraint violation. However, ECFDBO demonstrates the optimal overall planning trend, effectively bypassing obstacles while remaining closer to the terrain. It can be seen from the top view that ECFDBO’s path shows a reasonable “S”-shaped smooth turn when crossing obstacle gaps, with continuous inflection points and uniform curvature. GODBO’s planned path is suboptimal, exhibiting slight zigzagging in the same region and significantly violating altitude constraints, indicating insufficient step-size control during local searches. In contrast, DBO generates a significantly longer detour, characterized by sharp bends near multiple obstacles and a less smooth and compact path. This indicates that the original DBO tends to produce oscillations near obstacles as environmental complexity increases. In contrast, ECFDBO effectively avoids over-corrected paths due to its chaotic disturbance and refined control through a nonlinear scaling strategy. In Figure 10b, ECFDBO still maintains good altitude. Therefore, ECFDBO not only converges faster in Scenario 2 but also plans a lower cost and smoother path.

As the terrain in Scenario 3 becomes more complex, the requirements for global exploration and fine obstacle avoidance are higher. The convergence curve (Figure 7c) shows that ECFDBO still maintains the fastest convergence rate and approaches the optimal solution at about 100 generations. The fitness curves of GODBO and DBO remain significantly higher than that of ECFDBO in the whole iteration process, indicating that they fall into local optimization. It is obvious that ECFDBO has a more stable optimization ability and global convergence effect in a complex terrain environment. From the path shape (Figure 8c), the three algorithms successfully plan the path through the complex terrain, but the differences are significant: ECFDBO’s track avoidance strategy is more intelligent, and it starts to adjust the altitude and direction before entering the narrow channel to achieve a high-quality solution; GODBO and DBO take a more conservative approach; their paths are close to the edge of the terrain, resulting in a ‘sliding along the wall’ phenomenon. The top view (Figure 9c) clearly shows that ECFDBO’s path maintains a more uniform buffer distance from obstacle boundaries, while GODBO’s path even exhibits dangerous behavior. ECFDBO adopts intelligent boundary handling, and when particles approach the boundary, they are guided by a gradient to actively stay away from the “danger wall,” avoiding the “collision–retreat–re-collision” circuitous phenomenon of traditional DBO. In addition, the ECFDBO track turns more smoothly, with only necessary direction changes and no obvious back-and-forth twists; in contrast, the path generated by DBO contains several small loops, indicating that its particles were trapped in local search areas and missed better paths.

Scenario 4 adds safety constraints to the complex scenario, including multiple adjacent obstacles. A comparison of algorithm convergence (Figure 7d) shows that although the problem in this scenario is complex, ECFDBO can still converge to the vicinity of global optimization within about 120 generations and obtain the lowest path cost; GODBO converges to the final solution at a slightly higher cost; the DBO convergence curve stays at a higher cost value for a long time, showing an obvious premature convergence tendency and only obtaining suboptimal solutions. From a three-dimensional perspective (Figure 8d), the path planning results of each algorithm can be visually compared. ECFDBO successfully opens up a nearly direct path while avoiding obstacle clusters: when approaching complex obstacle clusters, the path does not plunge into crowded areas but chooses a relatively open detour corridor in advance and then bypasses obstacle clusters with smooth curves. This “round-before-through” strategy makes the ECFDBO path compact and efficient both in the top plane (Figure 9d) and in the height profile (Figure 10d): the curved path closely follows the globally optimal ideal path, with little excess detour and height fluctuation. In contrast, GODBO’s path has many sharp turns in this complex environment: from the top view, its path has made sharp turns close to right angles in order to avoid some obstacles, which not only increases the range but also causes the path not to be smooth; more seriously, it can be found in Figure 8d that GODBO’s planned path even has a “knotting” phenomenon. The path quality of the original DBO is the worst: DBO falls into local search in front of the obstacle group due to a lack of an effective disturbance-jumping mechanism, and finally, the planned track takes a large circle to reach the target, and the path cost is much higher than ECFDBO. ECFDBO stands out precisely because of its environment-aware boundary strategy: when particles approach an obstacle boundary, the algorithm does not simply truncate their coordinates but uses gradient information to bounce them away from the obstacle by a certain distance. This mechanism reduces the detour of repeated correction after collision with obstacles so that the ECFDBO track can keep smooth progress in complex areas. To sum up, ECFDBO achieves global optimal planning with the shortest path length, the lowest total cost, and the smoothest and safest path in the most complex Scenario 4, which fully proves the value of the improved strategy proposed in this paper in practical complex path planning.

In the above experiments in 3D environments of varying difficulty, the ECFDBO algorithm is able to converge to points with lower path generation values in all scenarios. Especially in complex environments, the advantages of ECFDBO over the original DBO and GODBO are more obvious—its global exploration capability ensures that the algorithm does not fall into suboptimal solutions in localized regions of obstacles, while the intelligent boundary handling and chaotic perturbation mechanisms improve path smoothing and reduce ineffective detours. The final results show that ECFDBO is able to more reliably plan safe and efficient 3D UAV paths, meeting the path optimality and smoothness requirements for practical tasks.

## 7. Conclusions

The Environment-aware Chaotic Force-field Dung Beetle Optimizer (ECFDBO) proposed in this paper significantly enhances the global optimization capability and convergence accuracy compared with the original DBO algorithm by employing three innovative strategies. Furthermore, ECFDBO is applied to plan remote sensing application paths in different complex environment scenarios. The results show that the ECFDBO algorithm can find the global optimal path in a complex environment with a faster convergence speed. These studies show that ECFDBO has broad prospects in the field of high-dimensional global optimization and can provide more efficient and reliable solutions for the autonomous navigation of unmanned aerial vehicles in complex environments. Nowadays, machine learning algorithms, such as reinforcement learning, are widely used for bionic robot path planning. Lin and Li et al. proposed a novel fixed-horizon constrained reinforcement learning (RL) framework [52] to ensure vehicle safety and strike a balance between accomplishing goals and providing comfort. Additionally, they proposed a sample efficient teacher-advice mechanism with Gaussian process (TAG) [53] to address the challenges of low sample efficiency and exploration difficulties in reinforcement learning. The above methods may be used for UAV path planning in the future.

## Figures and Tables

**Table 1:** Parameter settings.

Algorithm	Parameter
ECFDBO	$P_{p}$ $=0.2,\;\eta_{0}$ $=0.02,\;w_{0}$ $=0.5,\;p_{m}$ = 0.07
BWO	$W_{f}$ = 0.1, κ = (1 − 0.5 × t/iter)× rand, α = 3/2, $K_{d}$ = 0.05
COA	No internal hyperparameters
PIO	$N_{c1}$$=\mathrm{round}\;(0.7\times {\mathrm{Max}}_{\mathrm{iter}}),\;N_{c2}$ = Max_iter_ − Nc1
BOA	p = 0.8, a = 0.1, c = 0.01
NOA	α $=0.05,\;P_{a2}$ $=0.2,\;P_{rb}$ = 0.2
GODBO	$P_{p}$ = 0.2, λ = 1.25
DBO	$P_{p}$= 0.2

**Table 2:** F1–F30 Benchmark Function Test Results (dim = 30).

		ECFDBO	BWO	COA	PIO	BOA	NOA	GODBO	DBO
F1	mean	**63,942.51**	5.3 × 10^10^	6.01 × 10^10^	2.39 × 10^10^	5.61 × 10^10^	6.94 × 10^10^	70,930,059	2.64 × 10^8^
	std	**25,681.63**	3.89 × 10^9^	7.05 × 10^9^	4 × 10^9^	8.45 × 10^9^	7.01 × 10^9^	91,756,810	1.52 × 10^8^
	best	**26,987.47**	4.5 × 10^10^	4.35 × 10^10^	1.71 × 10^10^	3.7 × 10^10^	5.09 × 10^10^	98,021.64	8,135,863
	worst	**63,480.11**	5.37 × 10^10^	6.04 × 10^10^	2.31 × 10^10^	5.63 × 10^10^	6.86 × 10^10^	49,093,779	2.57 × 10^8^
	median	**136,598.9**	5.86 × 10^10^	7.33 × 10^10^	3.7 × 10^10^	7.02 × 10^10^	8.4 × 10^10^	4.57 × 10^8^	5.65 × 10^8^
F3	mean	**9633.591**	81,544.45	84,950.16	93,315.5	82,745.1	167,120.2	84,780.36	99,466.21
	std	**3718.314**	6400.38	5554.423	11,092.81	7651.117	18,639.5	9399.648	44,057.83
	best	**4364.107**	65,363.83	68,694.54	65,001.02	64,619.64	128,257.5	61,888.87	48,642.91
	worst	**9190.556**	82,800.58	86,511.15	94,228.91	83,836.86	167,579.1	87,999.2	88,573.77
	median	**20,079.23**	89,699.18	92,190.43	140,987.2	94,910.4	201,649.3	98,912.04	276,108.9
F4	mean	**507.3657**	12,623.19	15,825.34	2722.201	20,935.8	18,256.86	580.2727	661.3345
	std	**36.80775**	1638.889	2792.877	942.6409	3621.418	3024.038	78.66037	116.997
	best	**413.7077**	7410.967	7205.844	1384.752	13,918.25	11,069.96	473.5652	492.5788
	worst	**499.465**	12,837.3	16,777.87	2499.828	20,571.39	18,053.4	567.4128	640.5954
	median	**593.5448**	14,671.85	19,562.26	5098.445	26,488.42	23,726.71	842.6524	913.3457
F5	mean	800.4774	927.4693	913.1074	861.2371	912.0203	994.2789	**657.6842**	747.3314
	std	66.09734	**20.75242**	30.04247	35.8504	26.87911	30.76318	51.81988	46.88862
	best	689.4502	866.6334	859.8462	815.4365	861.6815	891.0154	**582.831**	670.1164
	worst	787.3878	928.829	913.4338	856.991	912.5462	1002.681	**644.9905**	749.2904
	median	941.7984	959.2968	979.8217	937.9318	957.8257	1033.27	**796.3756**	823.7392
F6	mean	656.6684	692.5882	692.5231	664.002	689.7923	700.5578	**632.6684**	651.2531
	std	10.41309	**3.796584**	6.053389	9.607602	6.46441	6.283517	7.125919	14.1612
	best	638.408	683.9351	680.0325	647.6615	672.0864	687.1371	**620.9715**	625.9021
	worst	656.0106	692.5493	692.5136	662.3847	690.1579	700.4147	**630.7543**	651.611
	median	682.1257	700.5901	706.2709	684.4867	698.8628	709.9328	**649.3964**	689.0801
F7	mean	1153.35	1399.737	1426.264	1480.295	1399.608	2509.026	**956.4392**	1033.291
	std	95.01219	**31.46096**	56.81814	69.98394	39.21844	146.9285	59.31968	96.73882
	best	984.1991	1324.88	1192.911	1319.496	1303.075	2124.928	**854.8254**	861.6912
	worst	1155.905	1405.592	1431.847	1488.556	1397.56	2510.022	**949.4535**	1023.429
	median	1363.504	1455.351	1492.507	1591.572	1475.421	2819.798	**1099.998**	1237.708
F8	mean	1025.492	1150.519	1144.503	1150.445	1140.776	1240.074	**956.0772**	1023.584
	std	55.48367	**17.49316**	26.30245	31.96821	22.10053	31.092	59.19539	53.63345
	best	932.3506	1115.986	1067.319	1051.653	1078.164	1152.245	**876.1469**	933.177
	worst	1017.925	1151.833	1147.954	1147.037	1146.534	1241.165	**938.4419**	1024.154
	median	1136.266	1196.125	1181.328	1205.558	1168.874	1295.581	**1083.507**	1126.276
F9	mean	9736.612	11,225.61	11,006.85	11,206.31	11,285.16	19,537.19	**3953.867**	6765.347
	std	2383.204	**1012.324**	1454.668	2377.326	1176.202	2469.75	1464.371	1543.686
	best	5041.098	8552.426	8263.224	7715.942	8045.321	13,774.64	**1759.752**	4228.991
	worst	9855.975	11,336.28	11,214.13	10,825.41	11,383.51	19,132.04	**3849.641**	6651.009
	median	15,743.02	13,329.95	13,500.85	18,884.46	13,336.9	23,146.73	**7918.796**	9490.701
F10	mean	**5713.745**	8901.387	8736.062	8948.604	9224.094	9078.285	6807.646	6231.936
	std	664.7464	357.743	476.0732	447.7387	346.0579	**310.5175**	1617.939	944.1829
	best	4282.283	8159.714	7632.897	7856.555	8399.939	7874.633	**3640.744**	4264.357
	worst	**5621.71**	8961.451	8780.951	8953.611	9296.556	9103.181	6538.687	5975.079
	median	**7289.91**	9538.599	9513.005	9625.635	9659.249	9465.218	9083.362	8844.381
F11	mean	**1292.336**	8200.144	9024.436	4832.391	9120.109	12,833.55	1401.827	1742.403
	std	**69.0754**	1516.37	1678.05	1121.911	2668.268	2871	142.0839	575.7978
	best	**1164.307**	4821.051	6320.424	2628.512	5682.718	5379.799	1234.603	1346.82
	worst	**1285.529**	8306.735	8685.513	4614.778	8221.468	13,228.65	1375.095	1613.581
	median	**1441.465**	11,521.07	12,781.11	7008.444	16,082.06	17,555.23	1911.36	4505.651
F12	mean	**2,519,837**	1.16 × 10^10^	1.3 × 10^10^	2.05 × 10^9^	1.33 × 10^10^	1 × 10^10^	1.05 × 10^8^	75,765,766
	std	**1,863,468**	2.18 × 10^9^	3.2 × 10^9^	6.39 × 10^8^	3.25 × 10^9^	2.26 × 10^9^	2.56 × 10^8^	1.19 × 10^8^
	best	**192,565.7**	8.3 × 10^9^	7.21 × 10^9^	8.47 × 10^8^	6.78 × 10^9^	5.96 × 10^9^	2,916,036	2,025,324
	worst	**2,047,460**	1.17 × 10^10^	1.28 × 10^10^	2.04 × 10^9^	1.31 × 10^10^	1.01 × 10^10^	20,653,606	27,026,788
	median	**8,109,615**	1.6 × 10^10^	1.96 × 10^10^	4.15 × 10^9^	2.14 × 10^10^	1.41 × 10^10^	1.09 × 10^9^	6.04 × 10^8^
F13	mean	**25,844.73**	6.55 × 10^9^	8.69 × 10^9^	6.77 × 10^8^	1.3 × 10^10^	5.64 × 10^9^	1.15 × 10^8^	11,920,817
	std	**20,870.87**	2.1 × 10^9^	4.36 × 10^9^	2.49 × 10^8^	6.3 × 10^9^	1.5 × 10^9^	6.03 × 10^8^	20,468,936
	best	**3899.699**	2.83 × 10^9^	2.84 × 10^9^	1.85 × 10^8^	3.88 × 10^9^	2.1 × 10^9^	41,537.44	46,900.35
	worst	**18,980.97**	6.51 × 10^9^	7.8 × 10^9^	6.19 × 10^8^	1.22 × 10^10^	5.79 × 10^9^	224,277.8	1,574,382
	median	**72,569.17**	1.05 × 10^10^	2.02 × 10^10^	1.22 × 10^9^	2.83 × 10^10^	9.46 × 10^9^	3.31 × 10^9^	71,958,593
F14	mean	**32,334.97**	4,257,854	3,960,435	815,745.2	4,297,723	3,025,255	283,592.4	209,998.4
	std	**26,512.96**	2,620,007	3,167,737	583,094.9	4,097,839	1,467,041	382,286.9	185,649
	best	**2309.498**	592,077.2	557,713	90,371.21	144,606.2	667,300.7	10,666.78	10,345.24
	worst	**25,730.5**	3,906,692	3,948,395	681,304.7	2,568,498	2,860,144	136,500.5	166,456.7
	median	**89,428.44**	14,500,690	14,945,409	2,395,542	19,757,277	7,075,432	1,673,009	881,024.9
F15	mean	**13,198.82**	3.28 × 10^8^	8.58 × 10^8^	1.52 × 10^8^	5.68 × 10^8^	8.21 × 10^8^	65,525.93	285,280.7
	std	**12,461.62**	1.9 × 10^8^	5.73 × 10^8^	91,543,844	5.76 × 10^8^	4.38 × 10^8^	53,997.25	1,102,114
	best	**1908.749**	13,466,524	45,179,493	34,061,391	37,393,246	1.09 × 10^8^	2920.75	7399.019
	worst	**9093.7**	3.34 × 10^8^	7.62 × 10^8^	1.13 × 10^8^	3.63 × 10^8^	8.15 × 10^8^	58,370.11	59,707.92
	median	**43,755.35**	8.69 × 10^8^	2.91 × 10^9^	4.03 × 10^8^	2.37 × 10^9^	1.71 × 10^9^	177,445.2	6,109,350
F16	mean	**2879.769**	5819.178	6364.759	4098.702	7467.678	5202.702	3115.283	3288.972
	std	**344.4561**	410.5533	906.3894	348.0564	1430.561	352.1852	420.6608	435.3645
	best	2175.863	4673.785	5161.759	3114.32	4054.969	4353.857	2333.674	**2172.459**
	worst	**2899.125**	5900.782	6260.549	4115.118	7507.335	5263.261	3055.136	3317.381
	median	**3567.785**	6616.584	8789.34	4960.323	9887.666	5749.903	4083.444	4184.224
F17	mean	**2516.491**	4475.714	5249.251	2971.063	10974.59	3581.037	2582.441	2679.382
	std	262.9417	763.2336	3076.794	**154.6144**	11,398.86	228.1887	289.5168	272.8822
	best	2054.322	3156.685	3006.496	2702.383	3899.054	3086.8	**1904.785**	2203.731
	worst	**2571.811**	4347.132	3900.741	2999.47	7324.974	3562.245	2597.015	2706.697
	median	**3159.862**	6487.527	17,638.14	3241.072	59,337.9	4019.663	3276.592	3195.838
F18	mean	**715,402.3**	54,249,832	59,452,992	11,870,742	49,325,991	40,892,718	1,948,317	4,033,524
	std	**817,243.3**	27,073,426	53,818,539	7,421,462	44,586,033	19,512,922	3,992,745	5,215,681
	best	41,453.47	6,640,996	1,889,149	2,794,686	6,903,898	5,674,777	**35,531.39**	78,313.29
	worst	**486,623.9**	53,683,497	49,593,129	10,764,934	31,256,161	39,061,988	860,592.8	1,839,512
	median	**3,439,557**	1.12 × 10^8^	2.11 × 10^8^	30,407,745	1.86 × 10^8^	1.06 × 10^8^	20,309,921	23,672,496
F19	mean	**21,262.95**	4.85 × 10^8^	7.77 × 10^8^	2 × 10^8^	6.64 × 10^8^	1.17 × 10^9^	4,097,332	5,784,654
	std	**18,941.24**	2.03 × 10^8^	5.22 × 10^8^	96,177,925	4.79 × 10^8^	4.63 × 10^8^	11,501,863	18,081,030
	best	2583.758	92,132,654	59,577,484	40,201,933	89,876,191	2.88 × 10^8^	**2193.569**	2638.476
	worst	**16,050.65**	4.87 × 10^8^	7.96 × 10^8^	2.14 × 10^8^	6.08 × 10^8^	1.22 × 10^9^	116,303.4	404,450.8
	median	**56,775.43**	9.58 × 10^8^	2.71 × 10^9^	4.2 × 10^8^	2.24 × 10^9^	1.97 × 10^9^	50,617,202	98,073,607
F20	mean	**2656.364**	3033.38	3094.954	3036.89	3118.8	3100.258	2697.781	2742.864
	std	239.1146	130.2307	168.8114	119.101	**92.77931**	110.8192	226.7765	260.9707
	best	2272.189	2771.009	2554.983	2787.174	2897.134	2857.476	2300.289	**2235.967**
	worst	2647.291	3043.592	3139.187	3045.243	3119.75	3110.219	**2628.047**	2707.018
	median	**3128.032**	3296.213	3339.509	3352.285	3288.163	3338.99	3137.841	3249.261
F21	mean	2580.126	2723.701	2747.425	2615.562	2751.371	2754.239	**2465.95**	2561.324
	std	73.88951	48.44362	45.47191	27.57139	48.36754	**23.98249**	45.84667	48.4529
	best	2447.708	2517.947	2648.155	2564.386	2623.924	2706.618	**2377.878**	2471.764
	worst	2572.032	2732.73	2752.298	2617.724	2755.723	2760.237	**2468.811**	2558.892
	median	2731.755	2792.62	2855.607	2663.135	2825.202	2790.236	**2560.656**	2655.951
F22	mean	6787.768	8871.049	9576.702	5759.646	7151.263	9518.539	**4205.682**	6215.286
	std	1677.095	**484.1969**	764.6035	2399.439	1026.057	723.9233	2613.766	2311.638
	best	**2303.192**	7887.258	6769.96	3515.935	4850.228	7868.123	2311.7	2364.278
	worst	7116.831	8882.628	9874.276	4697.181	7126.808	9654.074	**2427.148**	7212.762
	median	**8901.377**	9708.593	10,688.6	10,792.8	9364.771	10,622.98	9568.586	9886.574
F23	mean	3014.155	3333.174	3651.941	2990.432	3575.073	3402.129	**2915.651**	3014.023
	std	97.72177	50.0228	148.0593	**29.88314**	187.2047	60.65244	112.0721	86.11765
	best	2787.788	3226.259	3351.357	2935.955	3025.996	3276.915	**2762.188**	2864.823
	worst	2996.712	3337.957	3632.102	2989.931	3596.07	3403.872	**2871.606**	3007.588
	median	3250.481	3430.545	3998.319	**3056.997**	3864.495	3505.623	3139.631	3177.673
F24	mean	3161.378	3613.339	3787.649	3158.17	4161.534	3645.135	**3113.082**	3182.059
	std	102.5177	79.48947	185.5656	**42.43335**	238.4944	75.61685	90.56529	75.59769
	best	**2965.589**	3496.282	3365.74	3082.772	3590.092	3434.83	2979.835	3044.666
	worst	3173.165	3596.364	3808.872	3159.029	4152.174	3653.606	**3097.128**	3162.987
	median	3327.024	3762.824	4200.671	**3240.074**	4590.274	3824.527	3310.4	3336.49
F25	mean	**2896.548**	4480.734	5323.274	4724.265	5704.686	8804.558	2923.458	2999.593
	std	**15.91772**	199.7949	350.8185	380.5895	641.2731	863.4685	30.39884	77.06749
	best	**2883.728**	3962.203	4410.293	4017.356	4641.95	7384.903	2885.076	2894.905
	worst	**2889.672**	4477.085	5326.115	4702.903	5546.177	8584.713	2924.395	2983.4
	median	**2950.292**	4847.75	5965.091	5495.495	7193.241	10803.07	3023.588	3210.79
F26	mean	7455.369	10,646.15	11,735.05	6895.655	11,882.12	11,251.12	**6034.232**	7158.672
	std	1340.177	**561.0557**	861.7151	959.6498	788.1217	822.3199	997.3035	785.1776
	best	**2816.789**	9133.756	9854.465	5176.728	10,765.61	9540.23	3676.833	5295.298
	worst	7586.724	10,689.68	11,804.83	7168.295	11,911.44	11,502.11	**5888.133**	7151.111
	median	9757.087	11,751.22	13,408.35	8646.077	13,352.94	12,632.54	8731.387	**8632.776**
F27	mean	**3290.302**	4034.996	4495.708	3404.736	4395.659	4132.721	3349.182	3347.978
	std	**45.08133**	151.9417	395.5547	55.45793	363.7561	122.9201	79.86542	70.67476
	best	**3213.545**	3652.449	3830.985	3293.771	3752.556	3903.359	3261.087	3249.935
	worst	**3272.556**	4059.076	4402.444	3403.038	4458.546	4138.376	3323.077	3336.538
	median	**3392.306**	4294.696	5635.099	3520.293	5352.941	4402.514	3634.885	3534.849
F28	mean	**3252.387**	6458.329	7659.126	4566.759	8163.217	7789.137	3352.866	3604.37
	std	**42.03031**	325.0748	701.5746	459.2043	500.8554	776.3482	75.75684	687.9095
	best	**3200.329**	5574.211	6126.061	4073.511	7047.834	6335.197	3265.205	3299.473
	worst	**3250.148**	6521.058	7650.086	4413.026	8244.752	7842.011	3324.336	3392.65
	median	**3373.517**	7105.451	8955.831	5831.231	9021.743	9020.772	3558.877	6272.067
F29	mean	4429.617	6895.76	8345.137	5101.263	13,156.87	6395.72	**4214.615**	4549.805
	std	271.7949	711.9064	1580.409	304.2742	6171.057	428.0731	**229.5287**	392.7875
	best	3947.653	5733.582	5557.795	4427.106	6837.421	5507.769	3817.121	**3800.398**
	worst	4455.078	6874.303	8501.48	5147.45	11,409.63	6436.563	**4261.45**	4548.292
	median	4972.681	8645.288	12397.94	5690.939	34917.82	7228.335	**4635.857**	5311.23
F30	mean	**48,500.77**	1.16 × 10^9^	1.78 × 10^9^	1.23 × 10^8^	1.22 × 10^9^	6.8 × 10^8^	1,780,797	7,762,810
	std	**41,110.94**	3.57 × 10^8^	1.19 × 10^9^	50,993,436	5.91 × 10^8^	2.22 × 10^8^	2,843,252	22,587,572
	best	**6351.341**	5.08 × 10^8^	1.04 × 10^8^	35,740,734	3.27 × 10^8^	2.09 × 10^8^	30,084.02	13,525.09
	worst	**31,118.47**	1.15 × 10^9^	1.35 × 10^9^	1.15 × 10^8^	1.1 × 10^9^	6.98 × 10^8^	570,931.4	1,081,845
	median	**151,815.9**	1.96 × 10^9^	5.45 × 10^9^	2.4 × 10^8^	2.48 × 10^9^	1.1 × 10^9^	10,298,184	1.23 × 10^8^

**Table 3:** F1–F30 Benchmark Function Test Results (dim = 50).

		ECFDBO	BWO	COA	PIO	BOA	NOA	GODBO	DBO
F1	mean	**4,780,258**	1.06 × 10^11^	1.09 × 10^11^	9.65 × 10^10^	1.09 × 10^11^	1.71 × 10^11^	1.18 × 10^9^	9.19 × 10^9^
	std	**2,359,295**	5.01 × 10^9^	9.86 × 10^9^	1.39 × 10^10^	7.88 × 10^9^	1.07 × 10^10^	7.03 × 10^8^	1.69 × 10^10^
	best	**1,512,934**	9.37 × 10^10^	8.91 × 10^10^	6.82 × 10^10^	9.01 × 10^10^	1.5 × 10^11^	4.25 × 10^8^	4.64 × 10^8^
	worst	**3,928,363**	1.06 × 10^11^	1.1 × 10^11^	9.83 × 10^10^	1.1 × 10^11^	1.73 × 10^11^	1 × 10^9^	3.77 × 10^9^
	median	**10,522,602**	1.15 × 10^11^	1.25 × 10^11^	1.22 × 10^11^	1.22 × 10^11^	1.94 × 10^11^	3.35 × 10^9^	7.07 × 10^10^
F3	mean	**87,516.16**	249,766.4	197,257.9	264,701.7	316,388.5	329,198.4	322,936.4	248,865.5
	std	32,122.38	36,977.68	**21,021.17**	29,715.68	149,694.8	33,740.95	58,986.33	54,829.01
	best	**36,759.7**	189,932.7	162,315.7	179,046.2	170,203	248,425.4	193,637.4	145,758.5
	worst	**73,523.75**	251,556.4	195,704.8	266,654.4	272,373.2	331,103.4	314,708	247,962.2
	median	**162,429.7**	351,854.8	238,114.7	311,972.1	865,964.3	382,355.9	513,354.7	393,175.2
F4	mean	**609.8426**	34,119.13	39,293.57	15,732.3	41,410.92	51,193.12	912.5862	1194.075
	std	**60.86762**	3216.901	5100.663	5187.062	3473.452	8197.951	159.1647	299.3094
	best	**489.1183**	22,722.15	30,096.17	7784.69	31,673.1	37,072.26	654.7685	663.9026
	worst	**617.0304**	34,459.92	39,746.09	14,700.99	40,911.51	53,136.07	878.393	1115.647
	median	**800.1924**	39,085.95	48,427.4	31,976.03	49,882.52	63,973.42	1351.225	1895.822
F5	mean	1023.086	1196.792	1194.138	1243.679	1176.389	1447.111	**831.1338**	995.0373
	std	111.8223	**18.2011**	32.05341	28.3418	26.20114	33.46087	81.35437	99.21457
	best	837.5148	1151.511	1134.758	1167.687	1104.998	1368.81	**700.8344**	821.8647
	worst	1044.548	1198.489	1197.412	1241.588	1184.553	1455.214	**817.1006**	1005.631
	median	1273.856	1221.935	1243.223	1313.445	1218.404	1507.044	**1044.84**	1153.54
F6	mean	673.1587	703.0555	702.3727	691.9153	705.7748	720.2816	**647.1692**	670.9763
	std	6.741626	**4.470917**	4.770088	9.732927	5.495881	4.579695	7.042482	9.649086
	best	659.9427	689.8744	687.6308	673.5214	686.885	708.4121	**634.3791**	643.4003
	worst	673.5805	703.072	704.0603	693.1564	705.456	720.9183	**647.4455**	670.9801
	median	688.3811	710.4015	707.9847	711.0534	715.5399	727.7279	**663.3866**	687.7654
F7	mean	1677.512	1989.602	2070.307	2113.629	2008.223	4750.685	**1335.724**	1401.825
	std	129.802	43.66188	**41.57328**	77.63079	52.39592	171.1507	181.3475	134.9587
	best	1415.056	1864.435	1960.993	1884.382	1841.49	4404.823	**1075.477**	1139.746
	worst	1692.121	1994.192	2074.903	2120.844	2001.787	4751.85	**1307.007**	1407.649
	median	1973.952	2069.746	2130.137	2199.236	2110.807	5066.757	1893.642	**1700.882**
F8	mean	1351.27	1512.373	1495.853	1563.182	1512.089	1749.039	**1169.113**	1279.896
	std	85.06685	**17.77129**	27.11427	36.59189	24.964	42.2352	124.1553	117.3652
	best	1132.023	1467.454	1448.889	1487.389	1436.219	1623.599	**1024.438**	1068.045
	worst	1332.628	1511.177	1496.799	1568.833	1512.149	1756.379	**1111.142**	1312.018
	median	1524.114	1545.217	1545.219	1624.462	1555.27	1810.891	**1443.049**	1485.358
F9	mean	26,315.19	39,713.01	39,487.92	43,652.74	40,148.12	64,453.51	**18,684.12**	27,981.46
	std	5710.865	2801.281	**2733.521**	6709.924	3165.292	6313.14	5930.056	7751.885
	best	14,120	30,240.19	34,025.13	29,168.84	30,667.48	44,678.95	**10,217.21**	14,544.16
	worst	26,145.71	40,060.17	39,455.94	43,719.75	40,167.54	65,672.73	**18,116.57**	27,353.29
	median	39,054.82	44,701.06	44,175.14	54,292.88	45,526.84	76,863.93	**33,371.21**	43,059.4
F10	mean	**9036.244**	15,023.68	15,408.3	15,529.49	15,601.81	15,607.16	12,851.33	11,442.55
	std	1028.188	463.9161	452.2438	447.2961	565.7118	**364.0358**	2916.489	2387.653
	best	**6960.834**	13,673.81	14,072.49	14,574.59	14,099.78	14,972.36	7474.548	8069.785
	worst	**9123.119**	15,059.99	15,410.71	15,589.28	15,670.62	15,660.5	14,533.69	11,116.4
	median	**10,696.68**	15,644.29	16,156.46	16,218.35	16,541.27	16,122.54	15,705.3	15,258.18
F11	mean	**1456.014**	22,646.18	25,955.2	16,038.51	24,384.56	37,752.18	3113.923	5406.151
	std	**101.1321**	1911.871	2606.749	4284.927	2959.744	6875.879	685.7545	4643.407
	best	**1307.773**	18,668.89	19,768.28	9414.711	15,229.5	19,129.47	1899.894	2176.385
	worst	**1438.399**	22,946.78	26,342.5	15,599.38	25,237.19	38,325.34	3226.607	3474.322
	median	**1667.795**	25,295.74	30,296.26	26,549.58	28,484.62	49,140.39	4584.315	19,830.63
F12	mean	**21,208,223**	6.3 × 10^10^	8.9 × 10^10^	1.41 × 10^10^	8.17 × 10^10^	6.76 × 10^10^	3.98 × 10^8^	9.58 × 10^8^
	std	**11,394,622**	1.05 × 10^10^	1.46 × 10^10^	2.63 × 10^9^	1.76 × 10^10^	9.25 × 10^9^	4.05 × 10^8^	8.38 × 10^8^
	best	**7,817,785**	3.66 × 10^10^	5.51 × 10^10^	8.37 × 10^9^	5.32 × 10^10^	4.61 × 10^10^	31,344,196	31,725,817
	worst	**19,125,871**	6.25 × 10^10^	9.14 × 10^10^	1.39 × 10^10^	8.61 × 10^10^	6.79 × 10^10^	2.88 × 10^8^	6.88 × 10^8^
	median	**48,400,214**	8.33 × 10^10^	1.13 × 10^11^	2.09 × 10^10^	1.13 × 10^11^	8.4 × 10^10^	1.75 × 10^9^	3.61 × 10^9^
F13	mean	**65,586.63**	4.12 × 10^10^	4.74 × 10^10^	4.37 × 10^9^	4.13 × 10^10^	2.85 × 10^10^	1.93 × 10^8^	1.61 × 10^8^
	std	**44,267.62**	8.42 × 10^9^	1.62 × 10^10^	1.31 × 10^9^	1.72 × 10^10^	4.54 × 10^9^	5.22 × 10^8^	2.28 × 10^8^
	best	**13,035.42**	2.71 × 10^10^	1.55 × 10^10^	1.89 × 10^9^	1.66 × 10^10^	1.92 × 10^10^	114,020.3	2,961,075
	worst	**51,812.64**	4.15 × 10^10^	4.68 × 10^10^	4.28 × 10^9^	3.65 × 10^10^	2.87 × 10^10^	22,893,078	65,134,665
	median	**144,709.6**	5.91 × 10^10^	8.47 × 10^10^	6.93 × 10^9^	7.87 × 10^10^	3.76 × 10^10^	2.57 × 10^9^	9.92 × 10^8^
F14	mean	**327,094.4**	70,972,878	1.21 × 10^8^	4,716,715	1.57 × 10^8^	31,301,009	3,210,835	4,034,978
	std	**206,097.4**	26,730,785	1.07 × 10^8^	2,287,260	1.16 × 10^8^	16,751,125	4,229,751	4,957,667
	best	**62,115.75**	10,693,642	5,875,508	1,233,774	16,131,250	9,128,897	304,722.2	148,710.7
	worst	**311,704.9**	71,552,201	79,985,985	4,621,592	1.2 × 10^8^	27,603,229	1,954,626	1,928,327
	median	**859,561.4**	1.44 × 10^8^	4.11 × 10^8^	12,215,340	4.41 × 10^8^	85,198,632	23,139,889	17,643,989
F15	mean	**20,076.14**	6.39 × 10^9^	8.42 × 10^9^	1.85 × 10^9^	9.56 × 10^9^	7.81 × 10^9^	1,194,628	99,311,999
	std	**12,505.08**	1.61 × 10^9^	3.71 × 10^9^	7.37 × 10^8^	2.88 × 10^9^	2.2 × 10^9^	4,993,285	3.24 × 10^8^
	best	**3435.915**	3.34 × 10^9^	2.86 × 10^9^	3.12 × 10^8^	3.54 × 10^9^	2.44 × 10^9^	16,137.75	38,951.08
	worst	**19,200.35**	6.16 × 10^9^	8.49 × 10^9^	1.85 × 10^9^	9.96 × 10^9^	8.04 × 10^9^	95,439.28	222,537.5
	median	**55,648.57**	9.84 × 10^9^	2.03 × 10^10^	3.18 × 10^9^	1.53 × 10^10^	1.1 × 10^10^	27,128,857	1.76 × 10^9^
F16	mean	**4149.21**	8913.883	10,551.97	6367.51	11,540.07	8841.368	4350.922	4647.712
	std	**449.7375**	713.0557	1734.323	517.7536	1392.449	475.2364	620.8001	733.0185
	best	**2885.242**	7250.674	7519.845	5428.665	8080.71	7966.537	3034.95	3140.566
	worst	**4161.454**	8945.439	10,219.52	6349.436	11,493.53	8814.252	4308.845	4797.013
	median	**4885.024**	10,160.04	14,156.68	7510.284	14,834.54	9721.609	5773.102	6039.062
F17	mean	3925.459	7852.056	12,419.1	5903.533	17,758.61	16,857.18	**3714.467**	4287.897
	std	400.7181	1469.263	9511.617	524.5195	11,094.1	7660.292	**376.9633**	575.6267
	best	3266.528	5184.302	4962.228	4893.874	6891.405	6954.543	**2801.898**	2809.991
	worst	3885.232	7429.907	10,080.37	5944.598	15,095.38	17,259.1	**3752.541**	4376.222
	median	4527.253	11,919.57	54,864.4	7012.83	47,128.53	43,756.89	**4383.183**	5328.389
F18	mean	**3,741,347**	1.58 × 10^8^	2.06 × 10^8^	56,514,657	1.8 × 10^8^	1.7 × 10^8^	8,681,295	10,596,914
	std	**2,325,907**	63,623,326	1.04 × 10^8^	25,410,815	92,081,400	53,349,251	9,155,939	9,242,248
	best	**723,232.6**	52,320,253	77,846,397	19,530,349	57,970,648	75,065,695	724,814.8	1,571,896
	worst	**3,132,272**	1.59 × 10^8^	1.82 × 10^8^	51,753,001	1.41 × 10^8^	1.72 × 10^8^	5,021,894	7,209,046
	median	**10,168,401**	3.36 × 10^8^	5.33 × 10^8^	1.11 × 10^8^	4.29 × 10^8^	2.91 × 10^8^	42,010,779	39,489,195
F19	mean	**22,177.84**	3.65 × 10^9^	4.7 × 10^9^	6.87 × 10^8^	4.44 × 10^9^	3.52 × 10^9^	3,340,464	8,448,320
	std	**15,848.02**	8.07 × 10^8^	1.74 × 10^9^	2.54 × 10^8^	1.8 × 10^9^	7.66 × 10^8^	5,014,682	10,142,526
	best	**2419.492**	1.99 × 10^9^	1 × 10^9^	2.68 × 10^8^	1.72 × 10^9^	1.14 × 10^9^	12,709.89	258,850.9
	worst	**23,607.7**	3.62 × 10^9^	4.73 × 10^9^	6.82 × 10^8^	3.92 × 10^9^	3.53 × 10^9^	1,037,733	4,918,391
	median	**44,662.61**	5.31 × 10^9^	7.28 × 10^9^	1.18 × 10^9^	8.35 × 10^9^	4.8 × 10^9^	19,438,061	39,488,873
F20	mean	**3644.941**	4157.516	4297.97	4367.142	4365.791	4512.71	3788.856	3724.264
	std	405.6563	182.891	195.1588	236.7496	218.3726	**156.6324**	432.4924	269.4832
	best	**2731.173**	3642.467	3861.911	3678.193	3747.012	4093.051	2791.965	3237.719
	worst	3730.817	4153.203	4339.08	4388.035	4407.163	4520.621	3857.83	**3697.314**
	median	4448.922	4476.917	4654.814	4731.953	4701.596	4894.467	4418.513	**4227.031**
F21	mean	2854.438	3211.012	3266.611	2971.814	3230.192	3246.853	**2689.216**	2836.455
	std	104.4249	40.49479	91.52014	48.47485	61.12815	**34.9707**	110.5395	77.75509
	best	2624.227	3095.647	3044.184	2876.981	3144.784	3169.299	**2492.846**	2695.025
	worst	2870.038	3215.319	3261.033	2972.478	3221.754	3253.185	**2665.487**	2831.948
	median	3047.414	3267.575	3445.266	3083.429	3357.7	3314.383	**2909.121**	2953.604
F22	mean	**10,998.8**	16,980.06	17,249.4	16,679.94	17,358.84	17,391.27	13,652.73	12,998.59
	std	840.0132	**408.6059**	515.1264	2123.499	786.7584	417.0635	2907.168	2632.685
	best	9175.088	16,194.08	16,400.35	**7408.293**	13,847.85	16,508.11	9287.273	8554.412
	worst	**10,922.91**	17,105.06	17,387.52	17,186.66	17,511.75	17,358.62	12,992.19	12,206.8
	median	**12,981.21**	17,617.41	18,030.33	18,131.47	18,188.65	18,118.71	17,712.53	17,233.74
F23	mean	3581.643	4128.582	4620.601	3510.33	4719.551	4298.04	**3307.936**	3534.21
	std	206.2312	91.56528	189.0447	**68.58512**	192.4512	124.7457	129.4303	133.5617
	best	3210.139	3939.255	4212.774	3411.791	4076.143	4022.329	**3161.893**	3350.576
	worst	3583.159	4138.696	4653.107	3497.276	4730.426	4302.796	**3268.264**	3537.664
	median	3952.577	4266.704	4987.475	3681.977	5042.447	4499.2	**3650.281**	3956.54
F24	mean	3731.615	4570.291	4977.045	3600.492	5383.01	4623.296	**3516.178**	3755.547
	std	182.5002	145.2558	319.1224	**58.59646**	363.3184	151.54	256.9348	134.312
	best	3515.322	4265.015	4471.506	3480.267	4864.839	4229.267	**3166.45**	3428.635
	worst	3694.558	4581.483	4947.526	3600.111	5382.951	4625.649	**3453.59**	3798.043
	median	4218.938	4826.476	5813.129	**3707.121**	6419.906	4884.136	4227.354	3985.79
F25	mean	**3110.898**	14,313.62	15,991.59	14,126.02	16,062.94	33,891.03	3248.244	4482.356
	std	**35.02985**	642.6781	1098.787	2319.172	1040.241	2965.027	78.15422	2045.493
	best	**3039.404**	12,625.6	13,102.36	8969.727	13,429.07	24,792.33	3123.983	3140.849
	worst	**3115.631**	14,328.07	15,887.54	14,173.48	16,125.58	34,648.34	3243.365	3573.945
	median	**3192.159**	15,495.95	17,971.18	18,126.43	18,101.86	38,039.97	3434.72	10,113.06
F26	mean	12,568.05	16,850.6	17,622.64	17,458.21	17,780.67	21,232.97	**7701.159**	10,970.81
	std	1580.866	**378.2373**	535.129	2077.685	615.3835	1315.065	2403.614	1118.15
	best	9295.756	16,187.63	16,391.97	11,932.34	15,771.89	18,804.74	**4126.587**	8835.321
	worst	12,811.02	16,858.82	17,703.9	17,938.19	17,869.69	21,437.78	**7611.067**	11,041.12
	median	17,119.85	17,673.45	18,813.64	19,701.78	18,878.72	23,754.6	**12,024.9**	13,645.62
F27	mean	**3808.675**	6063.492	7074.102	4337.961	6850.284	6255.282	4020.108	3984.485
	std	235.8524	455.9033	910.8321	**196.7503**	807.757	321.4446	209.8758	213.1498
	best	**3484.347**	5243.028	5646.505	4030.389	5131.224	5697.883	3657.32	3623.358
	worst	**3811.76**	6093.332	6923.087	4318.229	6767.458	6244.369	4020.047	3939.278
	median	**4270.054**	6871.226	8560.403	4815.071	8550.204	6939.098	4531.801	4417.38
F28	mean	**3365.325**	12,358.22	14,036.87	9989.373	14,426.55	15,690.86	3964.528	6578.806
	std	**37.49875**	722.2068	1214.651	1047.597	1115.615	1440.892	1197.622	2123.016
	best	**3298.155**	9369.051	12,230.38	7433.328	12,290.5	12,654.15	3371.988	3527.566
	worst	**3369.195**	12,457.72	13,653.71	9811.273	14,322.91	15,661.53	3764.248	6543.57
	median	**3456.389**	13,161.69	16,948.66	12,382.35	17,099.86	18,663.63	10,185.55	10,123.58
F29	mean	**5497.258**	29,545.38	156,637.6	8103.622	309,734.5	25,633.53	5625.994	6369.865
	std	**590.1981**	17,812.2	192,179.3	635.5026	341,597	9502.369	632.0883	889.2101
	best	**4439.055**	10,118.99	25,201.37	6565.725	28,361.95	13,564.98	4492.346	4770.456
	worst	**5484.536**	25,437.29	79,776.49	8036.524	144,237.5	23,549.28	5650.587	6220.656
	median	7052.816	75,688.88	999,331.1	9552.535	1,209,936	55,501.01	**6680.621**	9653.24
F30	mean	**2,795,529**	5.14 × 10^9^	8.39 × 10^9^	1.2 × 10^9^	7.06 × 10^9^	5.73 × 10^9^	29,677,057	48,080,697
	std	**1,351,722**	1.11 × 10^9^	3.87 × 10^9^	3.56 × 10^8^	2.87 × 10^9^	1.39 × 10^9^	29,781,941	41,573,944
	best	**903,013.9**	2.54 × 10^9^	2.27 × 10^9^	6.3 × 10^8^	1.76 × 10^9^	2.99 × 10^9^	4,194,733	3,665,377
	worst	**2,509,618**	5.03 × 10^9^	7.53 × 10^9^	1.12 × 10^9^	7.05 × 10^9^	5.69 × 10^9^	15,930,412	26,709,720
	median	**6,397,697**	7.53 × 10^9^	1.68 × 10^10^	2.14 × 10^9^	1.23 × 10^10^	7.88 × 10^9^	1.02 × 10^8^	1.49 × 10^8^

**Table 4:** F1–F30 Benchmark Function Test Results (dim = 100).

		ECFDBO	BWO	COA	PIO	BOA	NOA	GODBO	DBO
F1	mean	**7.45 × 10^8^**	2.59 × 10^11^	2.73 × 10^11^	2.69 × 10^11^	2.61 × 10^11^	4.84 × 10^11^	3.7 × 10^10^	8.06 × 10^10^
	std	**3.24 × 10^8^**	5.57 × 10^9^	9.06 × 10^9^	1.3 × 10^10^	1.46 × 10^10^	1.66 × 10^10^	1.34 × 10^10^	6.3 × 10^10^
	best	**2.28 × 10^8^**	2.46 × 10^11^	2.54 × 10^11^	2.33 × 10^11^	2.27 × 10^11^	4.41 × 10^11^	1.82 × 10^10^	2.93 × 10^10^
	worst	**6.31 × 10^8^**	2.6 × 10^11^	2.75 × 10^11^	2.7 × 10^11^	2.62 × 10^11^	4.84 × 10^11^	3.61 × 10^10^	4.57 × 10^10^
	median	**1.61 × 10^9^**	2.68 × 10^11^	2.88 × 10^11^	2.91 × 10^11^	2.84 × 10^11^	5.17 × 10^11^	6.51 × 10^10^	2.5 × 10^11^
F3	mean	556,312.7	377,040	**351,483**	475,644.8	511,431.5	783,361.5	414,625.3	595,729.4
	std	155,549.4	50,453.21	**16,245.01**	176,121	174,033.4	87,093.05	106,216.4	250,553.3
	best	**284,156.4**	332,419.6	310,093.1	366,642.5	359,050.2	608,466.1	362,163.9	322,179.9
	worst	574,389.5	362,168.8	**356,070.1**	366,718	435,797	778,483.9	391,103.9	511,466.6
	median	964,242.4	560,682.7	**374,778.9**	883,061.5	947,707.4	975,420	951,696.2	1,369,806
F4	mean	**1103.319**	100,125.8	109,569.4	70,926.48	113,669.9	174,227.7	3727.137	19,936.42
	std	**109.6269**	7489.947	11,451.92	16,469.75	10,194.13	14,779.12	1350.386	20,626.2
	best	**948.5583**	85,618.23	87,562.86	39,553.18	90,745.05	147,507.9	1956.474	3387.313
	worst	**1076.578**	101,194.6	108,947.3	72,477.39	115,869.6	174,697.2	3401.511	8784.596
	median	**1481.919**	110,236.8	126,735.7	100,351.8	130,039.5	198,696.7	9386.499	71,595.01
F5	mean	1744.406	2120.383	2130.764	2219.419	2091.949	2741.801	**1684.584**	1692.849
	std	294.6566	**22.99016**	35.44666	55.07385	50.9796	66.98955	226.6237	205.015
	best	1373.308	2049.402	2060.307	2109.29	1936.35	2556.87	**1284.559**	1344.777
	worst	1714.638	2123.877	2133.904	2223.298	2106.804	2756.277	1692.64	**1613.662**
	median	2269.237	2159.801	2191.759	2307.57	2145.858	2837.671	2112.396	**2048.001**
F6	mean	678.0402	712.5224	713.1258	717.9441	712.8578	740.7731	**665.6168**	683.7687
	std	8.109982	**2.330429**	3.682679	5.089617	3.313486	3.780802	9.107656	13.90364
	best	666.344	707.813	701.4139	707.4029	706.6868	729.5406	**650.24**	657.0897
	worst	677.4142	712.1859	713.4107	720.0742	713.603	741.2842	**664.9418**	685.7911
	median	**698.8027**	717.3	717.6363	725.4998	718.7438	747.9626	699.5684	707.3842
F7	mean	3293.137	3884.255	4032.22	4143.678	3944.478	11,069.35	**2753.51**	3015.819
	std	200.926	61.64376	**52.51027**	104.3898	69.1295	479.8582	202.3837	256.7895
	best	2614.124	3761.538	3921.182	3800.1	3804.844	9824.999	**2370.676**	2389.143
	worst	3309.688	3879.63	4026.939	4147.419	3950.498	11,177.42	**2698.88**	3022.455
	median	3736.915	3984.399	4133.647	4285.594	4083.88	11,755.91	**3180.337**	3526.901
F8	mean	2222.104	2599.935	2598.42	2663.825	2578.03	3117.185	**1903.055**	2153.67
	std	202.2045	**34.72153**	56.75434	48.33201	38.17371	73.77207	200.1381	254.3752
	best	1717.655	2526.253	2448.614	2550.778	2508.156	2964.277	**1645.144**	1722.929
	worst	2221.624	2602.339	2615.513	2667.069	2582.048	3115.349	**1838.554**	2236.892
	median	2620.501	2680.056	2705.458	2736.03	2661.689	3234.99	**2372.973**	2498.944
F9	mean	**60,455.35**	80,183.84	81,018.74	99,296.14	86,041.04	165,765.3	77,050.36	73,587.85
	std	18655	4820.338	4885.766	6903.58	**3395.674**	10,464.62	8062.067	12,965.47
	best	**27,080.32**	65,379.89	69,477.43	78,988.2	80,435.73	142,076.6	58,765.57	35,460.35
	worst	**60,669.35**	80,472.15	80,475.79	102,981.4	85,766.82	165,432.2	77,127.33	75,350.98
	median	103,171.6	**87,361.81**	91,129.78	107,492	92,480.88	185,227.9	89,292.5	94,984.11
F10	mean	**18,753.74**	32,411.89	32,823.04	33,026.71	33,190.84	33,492.99	31,613.64	28,054.6
	std	1731.832	578.5304	855.6955	632.246	635.5477	**566.0072**	2337.055	4967.889
	best	**15,986.87**	31,194.72	30,940.53	31,454.9	31,740.45	32,155.73	22,861.36	18,960.93
	worst	**18,962.75**	32,577.92	32,940.26	33,120.2	33,221.75	33,575.51	32,405.64	29,006.16
	median	**23,201.87**	33,521.24	34,038.48	34,043.18	34,294.1	34,400.95	33,363.61	33,813.22
F11	mean	**30,562.76**	375,307.3	251,568.9	234,786.9	435,013.7	361,867.1	278,041	229,312.4
	std	**6570.78**	79,545.43	66,685.19	40,111.28	200,693.6	58,812.15	76,660.37	54,000.11
	best	**19,355.85**	253,923.3	143,315.6	125,476.8	165,383.3	250,249.2	134,985.8	151,359.1
	worst	**29,760.43**	368,621.2	225,813.5	239,133.6	361,261.8	364,866.1	276,670.5	220,557.2
	median	**43,382**	516,893.2	437,470.1	319,686.3	991,459	463,055.7	478,738.9	335,987.1
F12	mean	**3.35 × 10^8^**	1.92 × 10^11^	2.03 × 10^11^	8.9 × 10^10^	1.91 × 10^11^	2.31 × 10^11^	3.21 × 10^9^	7.15 × 10^9^
	std	**1.47 × 10^8^**	1.23 × 10^10^	1.81 × 10^10^	1.77 × 10^10^	2.33 × 10^10^	2.12 × 10^10^	1.07 × 10^9^	2.48 × 10^9^
	best	**1.23 × 10^8^**	1.6 × 10^11^	1.65 × 10^11^	5.96 × 10^10^	1.39 × 10^11^	1.73 × 10^11^	1.71 × 10^9^	1.37 × 10^9^
	worst	**3.26 × 10^8^**	1.95 × 10^11^	2.04 × 10^11^	9.01 × 10^10^	1.94 × 10^11^	2.37 × 10^11^	2.84 × 10^9^	6.64 × 10^9^
	median	**6.28 × 10^8^**	2.07 × 10^11^	2.39 × 10^11^	1.33 × 10^11^	2.36 × 10^11^	2.62 × 10^11^	5.34 × 10^9^	1.52 × 10^10^
F13	mean	**699,034**	4.38 × 10^10^	4.9 × 10^10^	1.56 × 10^10^	4.62 × 10^10^	5.55 × 10^10^	1.01 × 10^8^	3.83 × 10^8^
	std	**1,404,644**	3.91 × 10^9^	5.34 × 10^9^	3.05 × 10^9^	5.96 × 10^9^	4.91 × 10^9^	1.46 × 10^8^	2.85 × 10^8^
	best	**38,284.6**	3.67 × 10^10^	3.65 × 10^10^	6.95 × 10^9^	3.2 × 10^10^	4.24 × 10^10^	195,268	79,403,252
	worst	**89,752.62**	4.46 × 10^10^	4.86 × 10^10^	1.53 × 10^10^	4.71 × 10^10^	5.63 × 10^10^	53,102,300	2.98 × 10^8^
	median	**4,738,688**	5.04 × 10^10^	6 × 10^10^	2.16 × 10^10^	5.63 × 10^10^	6.33 × 10^10^	6.02 × 10^8^	1.29 × 10^9^
F14	mean	**3,357,850**	85,212,469	1.02 × 10^8^	68,947,180	1.51 × 10^8^	1.76 × 10^8^	5,119,034	19,596,326
	std	**2,371,169**	29,998,080	34,349,981	22,965,475	91,411,206	43,582,925	3,686,515	14,389,273
	best	**942,121.9**	31,318,902	44,596,733	15,755,741	45,787,788	88,132,376	1,248,844	3,347,050
	worst	**2,366,674**	81,087,973	89,294,636	70,365,901	1.21 × 10^8^	1.76 × 10^8^	4,347,843	16,060,509
	median	**10,420,191**	1.57 × 10^8^	1.82 × 10^8^	1.41 × 10^8^	3.93 × 10^8^	2.66 × 10^8^	18,256,524	68,172,667
F15	mean	**78,930.7**	2.34 × 10^10^	2.51 × 10^10^	5.57 × 10^9^	2.41 × 10^10^	2.23 × 10^10^	13,072,258	61,298,556
	std	**219,072.8**	3.02 × 10^9^	4.33 × 10^9^	1.12 × 10^9^	5.12 × 10^9^	5.04 × 10^9^	21,948,861	79,951,870
	best	**12,611.07**	1.36 × 10^10^	1.51 × 10^10^	3.66 × 10^9^	1.27 × 10^10^	1.39 × 10^10^	52,749.1	406,195.4
	worst	**26,017.69**	2.42 × 10^10^	2.57 × 10^10^	5.56 × 10^9^	2.41 × 10^10^	2.24 × 10^10^	3,704,249	38,353,874
	median	**1,220,023**	2.84 × 10^10^	3.21 × 10^10^	8.21 × 10^9^	3.38 × 10^10^	2.96 × 10^10^	77,559,893	3.09 × 10^8^
F16	mean	**7420.488**	22,623.58	25,147.88	14,599.08	26,320.23	24,164.12	7951.092	8898.95
	std	1072.346	1437.168	3156.294	**843.7633**	2172.911	1644.561	1032.6	1284.48
	best	**5570.599**	19,049.66	19,358.85	12,878.89	19,940.45	19,856.09	5912.598	6313.505
	worst	**7379.487**	22,827.85	25,509.03	14,565.16	26,451.68	24,062.32	7705.267	8900.273
	median	**9546.879**	25,581.13	30,852.49	16,755.66	30,537.53	27,797.61	10,722.2	11,774.85
F17	mean	**6750.273**	5,120,658	11,568,266	19,556.08	18,571,373	4,215,614	8014.545	9561.856
	std	**607.155**	3,202,454	10,391,713	7302.894	12,013,187	2,973,714	1475.525	1472.375
	best	**5389.357**	1,038,674	719,282.4	11,665.35	431,620.6	253,709.6	5894.588	6670.773
	worst	**6734.364**	4,444,374	9,134,410	17,682.34	19,581,069	3,070,116	7672.809	9609.199
	median	**7765.744**	11,556,604	38,037,312	39,841.93	57,540,752	10,549,137	13,547.69	12,806.76
F18	mean	**7,110,332**	2.01 × 10^8^	3.2 × 10^8^	1.23 × 10^8^	2.6 × 10^8^	3.31 × 10^8^	13,751,662	31,038,738
	std	**2,779,528**	53,722,189	1.74 × 10^8^	33,787,632	1.06 × 10^8^	1.03 × 10^8^	8,773,563	21,182,188
	best	**2,437,016**	87,289,245	38,115,404	61,624,382	67,995,553	1.5 × 10^8^	2,827,917	5,159,165
	worst	**6,988,379**	1.97 × 10^8^	2.93 × 10^8^	1.18 × 10^8^	2.57 × 10^8^	3.23 × 10^8^	11,671,407	29,795,728
	median	**12,595,111**	3.26 × 10^8^	7.19 × 10^8^	1.98 × 10^8^	5.18 × 10^8^	5.3 × 10^8^	34,595,900	88,488,020
F19	mean	**161,629.1**	2.24 × 10^10^	2.72 × 10^10^	5.25 × 10^9^	2.62 × 10^10^	2.45 × 10^10^	23,478,427	82,678,768
	std	**150,792.2**	2.39 × 10^9^	4.2 × 10^9^	1.01 × 10^9^	3.76 × 10^9^	2.58 × 10^9^	18,582,036	71,810,474
	best	**17,306.4**	1.72 × 10^10^	2.03 × 10^10^	3.31 × 10^9^	1.84 × 10^10^	1.95 × 10^10^	235,256.2	1,656,899
	worst	**129,312**	2.26 × 10^10^	2.76 × 10^10^	5.24 × 10^9^	2.62 × 10^10^	2.42 × 10^10^	19,932,316	60,757,956
	median	**770,506.8**	2.66 × 10^10^	3.52 × 10^10^	6.7 × 10^9^	3.3 × 10^10^	3.02 × 10^10^	72,024,184	3.6 × 10^8^
F20	mean	**6336.572**	7815.554	7936.265	8023.333	8061.241	8370.703	7436.64	7428.492
	std	730.5444	237.0489	322.0247	365.498	302.5378	**229.1073**	532.3598	657.3067
	best	**4856.539**	7222.014	7237.683	7322.811	7465.892	7798.554	5956.323	6130.059
	worst	**6494.878**	7841.19	7978.25	8028.876	8092.769	8389.837	7606.706	7453.894
	median	**7347.281**	8218.618	8376.735	8735.5	8550.418	8715.894	8196.152	8526.579
F21	mean	4091.623	4753.532	5054.394	4138.902	4835.805	4827.72	**3545.287**	4007.131
	std	202.3991	117.0087	215.807	120.4908	219.0194	**101.6755**	164.3183	179.7956
	best	3706.605	4460.952	4449.178	3924.682	4400.545	4541.913	**3209.056**	3581.077
	worst	4139.599	4766.447	5106.211	4141.252	4823.088	4850.111	**3529.698**	3982.011
	median	4547.738	4954.546	5383.477	4358.611	5245.605	4975.327	**3936.363**	4318.997
F22	mean	**21,835.86**	34,804.59	35,405.42	35,512.35	35,802.49	35,768.79	30,883.39	29,201.22
	std	1578.262	499.9623	**454.6997**	673.7102	563.8343	648.3102	5023.191	4840.573
	best	**18,345.09**	33,725.21	34,380.09	33,911.83	34,512.62	34,051.68	21,219.49	21,274.95
	worst	**22,189.25**	34,807.9	35,414.25	35,587.18	35,893.79	35,799.74	33,186.59	28,547.36
	median	**24,528.11**	35,643.73	36,446.65	36,867.86	36,636.76	36,958.27	35,643.22	35,948.67
F23	mean	4522.377	6128.485	6706.252	4726.073	6666.565	6624.233	**4458.082**	4862.778
	std	227.661	206.8003	311.8774	**144.3862**	298.6917	208.8306	334.0317	183.4844
	best	4069.015	5697.221	5925.037	4526.84	6167.803	6166.97	**3866.858**	4401.4
	worst	4541.962	6160.689	6759.413	4682.834	6672.552	6635.941	**4401.469**	4880.059
	median	**4985.777**	6464.255	7374.691	5104.453	7176.801	7003.271	5053.456	5160.213
F24	mean	5857.826	9342.537	10,841.56	5860.021	11,614.26	10,528.28	**5506.501**	6235.145
	std	378.8069	367.4904	824.4215	**249.144**	1349.797	673.2516	544.6357	411.0967
	best	5095.272	8696.912	8963.08	5409.182	8609.48	9354.27	**4438.242**	5502.835
	worst	5875.381	9381.774	10,730.26	5886.524	11,526.34	10,473.02	**5462.246**	6121.079
	median	6719.068	10,217.76	12,762.6	**6386.001**	13,823.65	11,594.64	6438.321	7131.038
F25	mean	**3769.489**	27,856.39	29,869.98	29,312.02	30,582.81	85,464.15	5939.275	10,182.26
	std	**111.1321**	1252.448	2092.453	3198.363	1622.11	7145.521	722.1818	7108.135
	best	**3560.144**	25,277.26	26,005.69	21,133.68	27,172.64	66,896.08	4862.769	4884.121
	worst	**3761.928**	27,753.1	29,784.64	29,816.04	30,471.82	87,164.45	5813.634	7050.382
	median	**4054.049**	30,542.99	33,982.04	35,071.9	33,326.43	99,108.89	7745.367	30,475.37
F26	mean	31,009.52	50,936.92	53,737.98	43,732.13	57,798.14	63,818.11	**21,038.14**	26,878.31
	std	4110.581	**1176.966**	2254.596	8990.192	2389.712	4546.23	3950.96	3906.133
	best	21,423.14	48,574.34	48,509.41	30,908.45	54,023.07	56,471.55	**13,467.27**	19877.39
	worst	31,427.39	50,935.76	54,026.64	44,323.95	58,188.65	63,397.93	**21,286.16**	26,315.97
	median	37,732.05	53,129.96	57,818.87	58,175.37	61,513.28	74,691.44	**31,846.06**	33,541.61
F27	mean	**4049.225**	12,738.09	14,612.9	6633.679	15,466	12,099.81	4588.784	4665.642
	std	**237.415**	887.3229	1847.36	462.838	1183.631	821.7461	437.8651	558.4765
	best	**3622.841**	11,001.37	9956.104	5859.08	12,395.44	10027.23	3820.702	3734.622
	worst	**4011.685**	12,881.18	14,617.13	6603.605	15,331.31	12,476.57	4517.536	4706.624
	median	**4536.897**	14,634.92	18,357.8	7681.489	17,298.81	13,150.6	5615.708	6164.552
F28	mean	**3928.902**	27,579.2	30,966.01	32,360.48	37,253.33	53,180.92	6247.656	18,748.06
	std	**175.0026**	932.4787	1115.262	2089.106	1964.436	3257.289	1052.154	6524.976
	best	**3719.935**	25,065.01	28,118.09	25,031.75	33,693.31	44,544.13	4336.486	6136.359
	worst	**3912.315**	27,677.51	31,323.85	33,469.65	37,559.39	53,123.78	6163.077	20,628.16
	median	**4428.841**	29,593.04	32,652.56	34,467.5	40,517.02	58,373.92	9215.413	30,822.45
F29	mean	**8480.565**	452,072.2	705,440.1	36,007.73	920,606.3	856,033	9526.919	11,454.74
	std	1080.918	180,448.5	485,272.9	20,755.47	470,669.8	403,997.3	**898.0878**	1669.909
	best	**5932.02**	93,201.62	72,902.48	20,394.78	258,530.2	123,197.8	7963.642	8446.091
	worst	**8524.741**	435,448.8	582,095.4	30,908.54	882,911.5	898,083	9369.629	11,355.29
	median	**11,096.79**	718,608.2	2,136,675	129,997.7	2,325,186	2,057,818	11,796.08	15,180.8
F30	mean	**3,917,708**	3.94 × 10^10^	4.42 × 10^10^	7.01 × 10^9^	4.13 × 10^10^	3.86 × 10^10^	1.43 × 10^8^	2.89 × 10^8^
	std	**1,861,671**	5.48 × 10^9^	6.62 × 10^9^	1.1 × 10^9^	5.24 × 10^9^	4.95 × 10^9^	1.37 × 10^8^	1.37 × 10^8^
	best	**1,659,875**	2.29 × 10^10^	2.86 × 10^10^	4.93 × 10^9^	2.49 × 10^10^	2.63 × 10^10^	11,050,311	59,217,772
	worst	**3,252,883**	4.16 × 10^10^	4.43 × 10^10^	7.01 × 10^9^	4.18 × 10^10^	3.97 × 10^10^	91,981,143	2.85 × 10^8^
	median	**9,756,674**	4.64 × 10^10^	5.49 × 10^10^	9.38 × 10^9^	5.05 × 10^10^	4.56 × 10^10^	5.76 × 10^8^	6.86 × 10^8^

**Table 5:** Wilcoxon test (dim = 30).

Function	BWO vs. ECFDBO	COA vs. ECFDBO	PIO vs. ECFDBO	BOA vs. ECFDBO	NOA vs. ECFDBO	GODBO vs. ECFDBO	DBO vs. ECFDBO
F1	**3.02 × 10^−11^**	**3.02 × 10^−11^**	**3.02 × 10^−11^**	**3.02 × 10^−11^**	**3.02 × 10^−11^**	**4.08 × 10^−11^**	**3.02 × 10^−11^**
F3	**3.02 × 10^−11^**	**3.02 × 10^−11^**	**3.02 × 10^−11^**	**3.02 × 10^−11^**	**3.02 × 10^−11^**	**3.02 × 10^−11^**	**3.02 × 10^−11^**
F4	**3.02 × 10^−11^**	**3.02 × 10^−11^**	**3.02 × 10^−11^**	**3.02 × 10^−11^**	**3.02 × 10^−11^**	**1.25 × 10^−5^**	**1.01 × 10^−8^**
F5	**4.2 × 10^−10^**	**5.97 × 10^−9^**	**0.00062**	**5.97 × 10^−9^**	**4.08 × 10^−11^**	**1.55 × 10^−9^**	**0.001953**
F6	**3.02 × 10^−11^**	**3.34 × 10^−11^**	**0.007617**	**4.08 × 10^−11^**	**3.02 × 10^−11^**	**2.37 × 10^−10^**	0.082357
F7	**4.98 × 10^−11^**	**8.99 × 10^−11^**	**3.69 × 10^−11^**	**5.49 × 10^−11^**	**3.02 × 10^−11^**	**6.12 × 10^−10^**	**1.64 × 10^−5^**
F8	**7.39 × 10^−11^**	**2.87 × 10^−10^**	**3.16 × 10^−10^**	**2.61 × 10^−10^**	**3.02 × 10^−11^**	**5.27 × 10^−5^**	0.958731
F9	**0.000399**	**0.010763**	**0.039167**	**0.000526**	**3.69 × 10^−11^**	**1.96 × 10^−10^**	**1.11 × 10^−6^**
F10	**3.02 × 10^−11^**	**3.02 × 10^−11^**	**3.02 × 10^−11^**	**3.02 × 10^−11^**	**3.02 × 10^−11^**	**0.005828**	**0.021506**
F11	**3.02 × 10^−11^**	**3.02 × 10^−11^**	**3.02 × 10^−11^**	**3.02 × 10^−11^**	**3.02 × 10^−11^**	**0.000318**	**2.37 × 10^−10^**
F12	**3.02 × 10^−11^**	**3.02 × 10^−11^**	**3.02 × 10^−11^**	**3.02 × 10^−11^**	**3.02 × 10^−11^**	**5.57 × 10^−10^**	**7.38 × 10^−10^**
F13	**3.02 × 10^−11^**	**3.02 × 10^−11^**	**3.02 × 10^−11^**	**3.02 × 10^−11^**	**3.02 × 10^−11^**	**2.61 × 10^−10^**	**7.39 × 10^−11^**
F14	**3.02 × 10^−11^**	**3.02 × 10^−11^**	**3.02 × 10^−11^**	**3.02 × 10^−11^**	**3.02 × 10^−11^**	**7.69 × 10^−8^**	**1.47 × 10^−7^**
F15	**3.02 × 10^−11^**	**3.02 × 10^−11^**	**3.02 × 10^−11^**	**3.02 × 10^−11^**	**3.02 × 10^−11^**	**4.42 × 10^−6^**	**1.31 × 10^−8^**
F16	**3.02 × 10^−11^**	**3.02 × 10^−11^**	**7.39 × 10^−11^**	**3.02 × 10^−11^**	**3.02 × 10^−11^**	**0.030317**	**0.000168**
F17	**3.34 × 10^−11^**	**4.08 × 10^−11^**	**4.18 × 10^−9^**	**3.02 × 10^−11^**	**3.69 × 10^−11^**	0.332855	0.05012
F18	**3.02 × 10^−11^**	**3.69 × 10^−11^**	**3.69 × 10^−11^**	**3.02 × 10^−11^**	**3.02 × 10^−11^**	0.185767	**0.000149**
F19	**3.02 × 10^−11^**	**3.02 × 10^−11^**	**3.02 × 10^−11^**	**3.02 × 10^−11^**	**3.02 × 10^−11^**	**0.00238**	**9.06 × 10^−8^**
F20	**6.53 × 10^−8^**	**9.26 × 10^−9^**	**3.35 × 10^−8^**	**2.23 × 10^−9^**	**3.5 × 10^−9^**	0.53951	0.166866
F21	**3.2 × 10^−9^**	**3.82 × 10^−10^**	**0.016285**	**4.2 × 10^−10^**	**6.07 × 10^−11^**	**3.65 × 10^−8^**	0.3871
F22	**1.69 × 10^−9^**	**3.82 × 10^−10^**	**0.003501**	0.78446	**1.61 × 10^−10^**	**0.006377**	0.982307
F23	**3.69 × 10^−11^**	**3.02 × 10^−11^**	0.464273	**1.78 × 10^−10^**	**3.02 × 10^−11^**	**0.001058**	0.888303
F24	**3.02 × 10^−11^**	**3.02 × 10^−11^**	0.641424	**3.02 × 10^−11^**	**3.02 × 10^−11^**	0.070127	0.501144
F25	**3.02 × 10^−11^**	**3.02 × 10^−11^**	**3.02 × 10^−11^**	**3.02 × 10^−11^**	**3.02 × 10^−11^**	**3.59 × 10^−5^**	**2.87 × 10^−10^**
F26	**4.08 × 10^−11^**	**3.02 × 10^−11^**	**0.035137**	**3.02 × 10^−11^**	**4.08 × 10^−11^**	**8.29 × 10^−6^**	0.08771
F27	**3.02 × 10^−11^**	**3.02 × 10^−11^**	**3.5 × 10^−9^**	**3.02 × 10^−11^**	**3.02 × 10^−11^**	**0.000268**	**0.000812**
F28	**3.02 × 10^−11^**	**3.02 × 10^−11^**	**3.02 × 10^−11^**	**3.02 × 10^−11^**	**3.02 × 10^−11^**	**1.1 × 10^−8^**	**2.37 × 10^−10^**
F29	**3.02 × 10^−11^**	**3.02 × 10^−11^**	**1.69 × 10^−9^**	**3.02 × 10^−11^**	**3.02 × 10^−11^**	**0.003848**	0.251881
F30	**3.02 × 10^−11^**	**3.02 × 10^−11^**	**3.02 × 10^−11^**	**3.02 × 10^−11^**	**3.02 × 10^−11^**	**8.48 × 10^−9^**	**5.19 × 10^−7^**

**Table 6:** Wilcoxon test (dim = 50).

Function	BWO vs. ECFDBO	COA vs. ECFDBO	PIO vs. ECFDBO	BOA vs. ECFDBO	NOA vs. ECFDBO	GODBO vs. ECFDBO	DBO vs. ECFDBO
F1	**3.02 × 10^−11^**	**3.02 × 10^−11^**	**3.02 × 10^−11^**	**3.02 × 10^−11^**	**3.02 × 10^−11^**	**3.02 × 10^−11^**	**3.02 × 10^−11^**
F3	**3.02 × 10^−11^**	**3.34 × 10^−11^**	**3.02 × 10^−11^**	**3.02 × 10^−11^**	**3.02 × 10^−11^**	**3.02 × 10^−11^**	**3.69 × 10^−11^**
F4	**3.02 × 10^−11^**	**3.02 × 10^−11^**	**3.02 × 10^−11^**	**3.02 × 10^−11^**	**3.02 × 10^−11^**	**8.99 × 10^−11^**	**4.5 × 10^−11^**
F5	**8.1 × 10^−10^**	**3.82 × 10^−9^**	**3.82 × 10^−10^**	**8.48 × 10^−9^**	**3.02 × 10^−11^**	**1.31 × 10^−8^**	0.297272
F6	**3.02 × 10^−11^**	**3.34 × 10^−11^**	**6.52 × 10^−9^**	**3.34 × 10^−11^**	**3.02 × 10^−11^**	**3.69 × 10^−11^**	0.347828
F7	**7.39 × 10^−11^**	**3.34 × 10^−11^**	**3.34 × 10^−11^**	**6.07 × 10^−11^**	**3.02 × 10^−11^**	**7.12 × 10^−9^**	**2.02 × 10^−8^**
F8	**9.76 × 10^−10^**	**1.31 × 10^−8^**	**5.49 × 10^−11^**	**9.76 × 10^−10^**	**3.02 × 10^−11^**	**4.42 × 10^−6^**	0.059428
F9	**1.61 × 10^−10^**	**1.33 × 10^−10^**	**3.82 × 10^−10^**	**1.21 × 10^−10^**	**3.02 × 10^−11^**	**1.34 × 10^−5^**	0.379036
F10	**3.02 × 10^−11^**	**3.02 × 10^−11^**	**3.02 × 10^−11^**	**3.02 × 10^−11^**	**3.02 × 10^−11^**	**2.28 × 10^−5^**	**7.2 × 10^−5^**
F11	**3.02 × 10^−11^**	**3.02 × 10^−11^**	**3.02 × 10^−11^**	**3.02 × 10^−11^**	**3.02 × 10^−11^**	**3.02 × 10^−11^**	**3.02 × 10^−11^**
F12	**3.02 × 10^−11^**	**3.02 × 10^−11^**	**3.02 × 10^−11^**	**3.02 × 10^−11^**	**3.02 × 10^−11^**	**5.49 × 10^−11^**	**5.49 × 10^−11^**
F13	**3.02 × 10^−11^**	**3.02 × 10^−11^**	**3.02 × 10^−11^**	**3.02 × 10^−11^**	**3.02 × 10^−11^**	**8.99 × 10^−11^**	**3.02 × 10^−11^**
F14	**3.02 × 10^−11^**	**3.02 × 10^−11^**	**3.02 × 10^−11^**	**3.02 × 10^−11^**	**3.02 × 10^−11^**	**3.82 × 10^−10^**	**2.57 × 10^−7^**
F15	**3.02 × 10^−11^**	**3.02 × 10^−11^**	**3.02 × 10^−11^**	**3.02 × 10^−11^**	**3.02 × 10^−11^**	**1.55 × 10^−9^**	**6.07 × 10^−11^**
F16	**3.02 × 10^−11^**	**3.02 × 10^−11^**	**3.02 × 10^−11^**	**3.02 × 10^−11^**	**3.02 × 10^−11^**	0.1809	**0.004637**
F17	**3.02 × 10^−11^**	**3.02 × 10^−11^**	**3.02 × 10^−11^**	**3.02 × 10^−11^**	**3.02 × 10^−11^**	0.085	**0.002891**
F18	**3.02 × 10^−11^**	**3.02 × 10^−11^**	**3.02 × 10^−11^**	**3.02 × 10^−11^**	**3.02 × 10^−11^**	**0.015014**	**0.000284**
F19	**3.02 × 10^−11^**	**3.02 × 10^−11^**	**3.02 × 10^−11^**	**3.02 × 10^−11^**	**3.02 × 10^−11^**	**5.46 × 10^−9^**	**3.02 × 10^−11^**
F20	**1.87 × 10^−7^**	**1.1 × 10^−8^**	**5 × 10^−9^**	**4.57 × 10^−9^**	**1.78 × 10^−10^**	0.122353	0.589451
F21	**3.02 × 10^−11^**	**3.34 × 10^−11^**	**2.68 × 10^−6^**	**3.02 × 10^−11^**	**3.02 × 10^−11^**	**1.03 × 10^−6^**	0.347828
F22	**3.02 × 10^−11^**	**3.02 × 10^−11^**	**1.86 × 10^−9^**	**3.02 × 10^−11^**	**3.02 × 10^−11^**	**0.000526**	**0.002755**
F23	**3.69 × 10^−11^**	**3.02 × 10^−11^**	0.149449	**3.02 × 10^−11^**	**3.02 × 10^−11^**	**2 × 10^−6^**	0.283778
F24	**3.02 × 10^−11^**	**3.02 × 10^−11^**	**0.000587**	**3.02 × 10^−11^**	**3.02 × 10^−11^**	**0.000399**	0.149449
F25	**3.02 × 10^−11^**	**3.02 × 10^−11^**	**3.02 × 10^−11^**	**3.02 × 10^−11^**	**3.02 × 10^−11^**	**4.2 × 10^−10^**	**4.5 × 10^−11^**
F26	**2.87 × 10^−10^**	**4.98 × 10^−11^**	**9.26 × 10^−9^**	**4.08 × 10^−11^**	**3.02 × 10^−11^**	**2.03 × 10^−9^**	**4.08 × 10^−5^**
F27	**3.02 × 10^−11^**	**3.02 × 10^−11^**	**1.69 × 10^−9^**	**3.02 × 10^−11^**	**3.02 × 10^−11^**	**0.00077**	**0.004226**
F28	**3.02 × 10^−11^**	**3.02 × 10^−11^**	**3.02 × 10^−11^**	**3.02 × 10^−11^**	**3.02 × 10^−11^**	**2.15 × 10^−10^**	**3.02 × 10^−11^**
F29	**3.02 × 10^−11^**	**3.02 × 10^−11^**	**4.08 × 10^−11^**	**3.02 × 10^−11^**	**3.02 × 10^−11^**	0.290472	**7.74 × 10^−6^**
F30	**3.02 × 10^−11^**	**3.02 × 10^−11^**	**3.02 × 10^−11^**	**3.02 × 10^−11^**	**3.02 × 10^−11^**	**1.46 × 10^−10^**	**2.15 × 10^−10^**

**Table 7:** Wilcoxon test (dim = 100).

Function	BWO vs. ECFDBO	COA vs. ECFDBO	PIO vs. ECFDBO	BOA vs. ECFDBO	NOA vs. ECFDBO	GODBO vs. ECFDBO	DBO vs. ECFDBO
F1	**3.02 × 10^−11^**	**3.02 × 10^−11^**	**3.02 × 10^−11^**	**3.02 × 10^−11^**	**3.02 × 10^−11^**	**3.02 × 10^−11^**	**3.02 × 10^−11^**
F3	**7.22 × 10^−6^**	**6.53 × 10^−7^**	**0.046756**	0.162375	**4.69 × 10^−8^**	**0.000141**	0.994102
F4	**3.02 × 10^−11^**	**3.02 × 10^−11^**	**3.02 × 10^−11^**	**3.02 × 10^−11^**	**3.02 × 10^−11^**	**3.02 × 10^−11^**	**3.02 × 10^−11^**
F5	**6.28 × 10^−6^**	**4.12 × 10^−6^**	**6.01 × 10^−8^**	**1.09 × 10^−5^**	**3.02 × 10^−11^**	0.549327	0.761828
F6	**3.02 × 10^−11^**	**3.02 × 10^−11^**	**3.02 × 10^−11^**	**3.02 × 10^−11^**	**3.02 × 10^−11^**	**3.26 × 10^−7^**	0.082357
F7	**3.02 × 10^−11^**	**3.02 × 10^−11^**	**3.02 × 10^−11^**	**3.02 × 10^−11^**	**3.02 × 10^−11^**	**6.72 × 10^−10^**	**2.6 × 10^−5^**
F8	**1.41 × 10^−9^**	**1.55 × 10^−9^**	**8.15 × 10^−11^**	**5.46 × 10^−9^**	**3.02 × 10^−11^**	**2.15 × 10^−6^**	0.520145
F9	**3.26 × 10^−7^**	**2.2 × 10^−7^**	**2.61 × 10^−10^**	**8.48 × 10^−9^**	**3.02 × 10^−11^**	**2.6 × 10^−5^**	**0.001857**
F10	**3.02 × 10^−11^**	**3.02 × 10^−11^**	**3.02 × 10^−11^**	**3.02 × 10^−11^**	**3.02 × 10^−11^**	**3.34 × 10^−11^**	**4.62 × 10^−10^**
F11	**3.02 × 10^−11^**	**3.02 × 10^−11^**	**3.02 × 10^−11^**	**3.02 × 10^−11^**	**3.02 × 10^−11^**	**3.02 × 10^−11^**	**3.02 × 10^−11^**
F12	**3.02 × 10^−11^**	**3.02 × 10^−11^**	**3.02 × 10^−11^**	**3.02 × 10^−11^**	**3.02 × 10^−11^**	**3.02 × 10^−11^**	**3.02 × 10^−11^**
F13	**3.02 × 10^−11^**	**3.02 × 10^−11^**	**3.02 × 10^−11^**	**3.02 × 10^−11^**	**3.02 × 10^−11^**	**2.37 × 10^−10^**	**3.02 × 10^−11^**
F14	**3.02 × 10^−11^**	**3.02 × 10^−11^**	**3.02 × 10^−11^**	**3.02 × 10^−11^**	**3.02 × 10^−11^**	**0.029205**	**1.29 × 10^−9^**
F15	**3.02 × 10^−11^**	**3.02 × 10^−11^**	**3.02 × 10^−11^**	**3.02 × 10^−11^**	**3.02 × 10^−11^**	**3.47 × 10^−10^**	**3.34 × 10^−11^**
F16	**3.02 × 10^−11^**	**3.02 × 10^−11^**	**3.02 × 10^−11^**	**3.02 × 10^−11^**	**3.02 × 10^−11^**	0.051877	**6.77 × 10^−5^**
F17	**3.02 × 10^−11^**	**3.02 × 10^−11^**	**3.02 × 10^−11^**	**3.02 × 10^−11^**	**3.02 × 10^−11^**	**2.43 × 10^−5^**	**2.61 × 10^−10^**
F18	**3.02 × 10^−11^**	**3.02 × 10^−11^**	**3.02 × 10^−11^**	**3.02 × 10^−11^**	**3.02 × 10^−11^**	**0.000587**	**1.6 × 10^−7^**
F19	**3.02 × 10^−11^**	**3.02 × 10^−11^**	**3.02 × 10^−11^**	**3.02 × 10^−11^**	**3.02 × 10^−11^**	**4.5 × 10^−11^**	**3.02 × 10^−11^**
F20	**4.98 × 10^−11^**	**4.5 × 10^−11^**	**3.34 × 10^−11^**	**3.02 × 10^−11^**	**3.02 × 10^−11^**	**7.69 × 10^−8^**	**1.11 × 10^−6^**
F21	**3.34 × 10^−11^**	**3.34 × 10^−11^**	0.371077	**4.08 × 10^−11^**	**3.34 × 10^−11^**	**1.33 × 10^−10^**	0.111987
F22	**3.02 × 10^−11^**	**3.02 × 10^−11^**	**3.02 × 10^−11^**	**3.02 × 10^−11^**	**3.02 × 10^−11^**	**4.57 × 10^−9^**	**2.92 × 10^−9^**
F23	**3.02 × 10^−11^**	**3.02 × 10^−11^**	**0.000301**	**3.02 × 10^−11^**	**3.02 × 10^−11^**	0.411911	**6.05 × 10^−7^**
F24	**3.02 × 10^−11^**	**3.02 × 10^−11^**	0.982307	**3.02 × 10^−11^**	**3.02 × 10^−11^**	**0.011228**	**0.000952**
F25	**3.02 × 10^−11^**	**3.02 × 10^−11^**	**3.02 × 10^−11^**	**3.02 × 10^−11^**	**3.02 × 10^−11^**	**3.02 × 10^−11^**	**3.02 × 10^−11^**
F26	**3.02 × 10^−11^**	**3.02 × 10^−11^**	**9.06 × 10^−8^**	**3.02 × 10^−11^**	**3.02 × 10^−11^**	**3.2 × 10^−9^**	**0.000356**
F27	**3.02 × 10^−11^**	**3.02 × 10^−11^**	**3.02 × 10^−11^**	**3.02 × 10^−11^**	**3.02 × 10^−11^**	**8.2 × 10^−7^**	**3.32 × 10^−6^**
F28	**3.02 × 10^−11^**	**3.02 × 10^−11^**	**3.02 × 10^−11^**	**3.02 × 10^−11^**	**3.02 × 10^−11^**	**3.34 × 10^−11^**	**3.02 × 10^−11^**
F29	**3.02 × 10^−11^**	**3.02 × 10^−11^**	**3.02 × 10^−11^**	**3.02 × 10^−11^**	**3.02 × 10^−11^**	**0.000178**	**3.82 × 10^−9^**
F30	**3.02 × 10^−11^**	**3.02 × 10^−11^**	**3.02 × 10^−11^**	**3.02 × 10^−11^**	**3.02 × 10^−11^**	**3.02 × 10^−11^**	**3.02 × 10^−11^**

**Table 8:** Friedman test in different dimensions.

Test Functions and Dimensions	Algorithm and the Friedman Test								
Algorithm		ECFDBO	BWO	COA	PIO	BOA	NOA	GODBO	DBO
CEC2017-30D	Friedman	2.1241	5.2759	6.2828	4.1517	6.3724	6.3034	2.3655	3.1241
	Rankings	1	5	6	4	8	7	2	3
CEC2017-50D	Friedman	2.0276	4.8690	6.0690	4.4621	6.4345	6.4897	2.5379	3.1103
	Rankings	1	5	6	4	7	8	2	3
CEC2017-100D	Friedman	1.9931	4.6414	5.8483	4.6483	6.2207	6.8138	2.4621	3.3724
	Rankings	1	4	6	5	7	8	2	3

**Table 9:** Comparison of fitness values of various algorithms.

		DBO	BWO	COA	PIO	BOA	NOA	GODBO	ECFDBO
Scenario 1	mean	2.2316	1.5763	1.4832	2.0817	2.6013	2.7279	**1.2346**	1.5688
	best	1.4468	1.5651	1.4044	1.7332	1.9031	2.6730	**0.3057**	0.7681
Scenario 2	mean	2.4209	1.5498	3.7070	3.0094	12.9084	9.0949	2.4308	**1.5242**
	best	2.0802	1.4630	2.1446	1.8516	9.2956	6.6315	1.7541	**0.166** **0**
Scenario 3	mean	1.7941	1.7472	1.8963	**1.3048**	2.9077	2.4024	1.7808	1.3504
	best	1.4477	1.707	1.7199	0.9515	2.0745	1.8298	1.1031	**0.8294**
Scenario 4	mean	2.3057	1.7660	2.5658	3.4131	7.1357	7.5692	2.9202	**1.4131**
	best	2.0574	1.7369	2.2404	2.5915	4.5966	5.4114	2.1725	**0.8181**

## Data Availability

The original contributions presented in this study are included in the article. further inquiries can be directed to the corresponding author(s).

## Appendix A

This appendix section consists of a classical convergence curve for CEC2017 (dim = 30, 50, 100). Provides visual evidence for the basic function tests in Section 5, including typical single-peak functions F1/F3, simple multi-peak functions F5/F8/F9/F10, hybrid functions F15/F16/F17/F20, and composition functions F21/F23/F24/F27/F29/F30.
